# Habitat Restorations in an Urban Landscape Rapidly Assemble Diverse Pollinator Communities That Persist

**DOI:** 10.1111/ele.70037

**Published:** 2024-12-31

**Authors:** Jens Ulrich, Risa D. Sargent

**Affiliations:** ^1^ Department of Applied Biology University of British Columbia Vancouver British Columbia Canada; ^2^ Faculty of Land and Food Systems University of British Columbia Vancouver British Columbia Canada

**Keywords:** conservation, habitat restoration, metacommunity dynamics, network, occupancy models, pollination, pollinators, specialisation, urban ecology

## Abstract

Ecological restoration is a leading approach to mitigating biodiversity decline. While restoration often leads to an immediate increase in species abundance or diversity, it is rarely clear whether it supports longer‐term biodiversity gains at the landscape scale. To examine the impacts of urban restoration on pollinator biodiversity, we conducted a 3‐year natural experiment in 18 parks across a large metropolitan area. We applied an occupancy model to our survey data to determine how restoration, woody plant density and pollinator specialisation impacted interannual pollinator metacommunity dynamics. Restoration drove a rapid increase in pollinator species occurrence that was maintained through a positive balance between colonisation and persistence, resulting in pollinator species richness gains that are retained. We conclude that urban restoration can effectively conserve pollinator biodiversity by influencing the processes that underlie long‐term population stability. Our results highlight the need to study the long‐term effects of restoration in different landscape contexts.

## Graphical abstract

While restoration often leads to an immediate increase in abundance or diversity, it is rarely clear whether it supports longer‐term biodiversity gains at the landscape scale. Habitat restorations in an urban landscape drove a rapid increase in pollinator species occurrence that was maintained through a positive balance between colonisation and persistence, resulting in pollinator species richness gains that are retained. We conclude that urban restoration can effectively conserve pollinator biodiversity by influencing the processes that underlie long‐term population stability for many pollinator species.

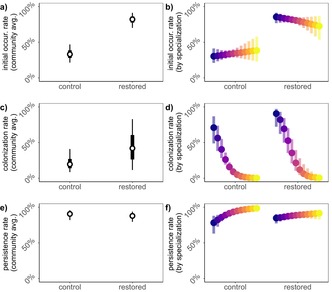

## 1 Introduction

Land use change is a critical threat to global biodiversity (IPBES 2019), driving biotic homogenisation and species loss (Devictor et al. 2008; Montràs‐Janer et al. 2024), with cascading impacts on ecosystem services (Connelly, Poveda, and Loeb 2015). Sixty percent of global land use change within the past century is attributable to human activity, largely through agricultural and urban expansion (Song et al. 2018). To mitigate the negative effects of land use change on biodiversity, there is growing interest in restoring habitat within human dominated lands (Kovács‐Hostyánszki et al. 2017). Understanding how to implement restorations that effectively support biodiversity is especially important for insect pollinators as these critical ecosystem players are facing widespread declines (Wagner et al. 2021).

It is well established that restoration—defined here as the process of taking areas degraded by human activity and returning them to a state that better supports wildlife (WWF Canada 2024)—increases pollinator biodiversity in the short term. In agricultural settings, hedgerows to restore flowering plant diversity are associated with higher pollinator abundance and diversity (Lowe, Groves, and Gratton 2021). Similarly, in urban environments, studies find links between plant restoration and pollinator biodiversity (Griffiths‐Lee, Nicholls, and Goulson 2022; Brown et al. 2024; Cloutier et al. 2024). However, most studies only provide snapshot measures of pollinator abundance and diversity; these could be misleading if restorations simply congregate pollinators that are already present in the landscape, but do not lead to longer‐term abundance and diversity gains (Tscharntke et al. 2012). Because few studies have tracked how populations change over space and time in response to restoration, it is currently unclear whether typical habitat restorations support biodiversity over the long term.

The long‐term value of restoration can be quantified by examining its impacts on the processes that give rise to biodiversity patterns (Chase et al. 2020). Across a network of habitat patches linked by dispersal, species occurrence (presence/absence in patches) fluctuates from year to year (Leibold et al. 2004). When species occur at more patches, their retention in the network of patches is more stable (Hanski 1999; Leibold et al. 2004). Restorations will increase overall occurrence rates for more species, and therefore long‐term, regional diversity, if they create a positive balance between the likelihood that species disperse into unoccupied habitat (i.e., colonisation) and the likelihood that species in occupied habitat are retained through time (i.e., persistence) (Chase et al. 2020).

Studies in agricultural landscapes demonstrate that restoration promotes long‐term pollinator biodiversity gains by increasing colonisation and persistence (M'Gonigle et al. 2015; Ponisio et al. 2019). However, it is currently unknown whether these findings translate from agriculture to urban landscapes. Unlike conventional agricultural contexts, urban landscapes offer heterogeneously spaced micro‐habitats such as private and public gardens that provide refuge from the pressures of urbanisation (Baldock et al. 2019). These interspersed habitats can harbour source populations that disperse to restored sites, or, alternatively, facilitate greater connectivity among sites (Driscoll et al. 2013; Tscharntke et al. 2012). Understanding how metacommunity dynamics respond to restoration in different landscapes is critical for designing practices that support long‐term biodiversity conservation.

In 2020, the City of Vancouver, Canada, introduced a series of habitat restorations in urban parks that consisted of reduced mowing alongside wildflower seeding. Taking advantage of this natural experiment, we surveyed wild bee and hoverfly pollinator communities for 3 years in restored and control parks. We used interaction data to determine the degree to which the pollinator species we surveyed tended to be dietary specialists on a few plant taxa versus generalists across many. Simultaneously, we measured the floral resources provided by existing woody plants in each park. We applied an occupancy model to our pollinator data that allowed us to test how restoration, woody plants and pollinator dietary specialisation affect the dynamics that underlie community biodiversity trajectories.

Based on previous studies, we predicted that restoration would be associated with an increase in the initial occurrence of pollinators and, additionally, that greater floral resources in restored parks would encourage additional species to colonise in subsequent years and raise the odds that they persist. We expected that additional food resources provided by existing flowering woody plants would boost initial occurrence and enhance colonisation and persistence of pollinators. We predicted that this effect would be especially pronounced for specialists, because of the higher probability that their preferred plant taxa would be present at sites with more woody and/or herbaceous plants (Ponisio et al. 2019). We synthesised our estimates for initial occurrence, colonisation and persistence to compare species richness trajectories in restored and control parks. Finally, to examine whether the restorations resulted in increased pollination services, we used *Clarkia amoena* as a ‘phytometer’ to conduct a pollen limitation experiment. By quantifying the effects of restoration on pollinator metacommunity dynamics and pollination services, we assessed whether restoration accomplishes the goal of long‐term plant–pollinator conservation in an urban setting.

## 2 Material and Methods

### 2.1 Data Collection

#### 2.1.1 Site Selection

**FIGURE 1:**
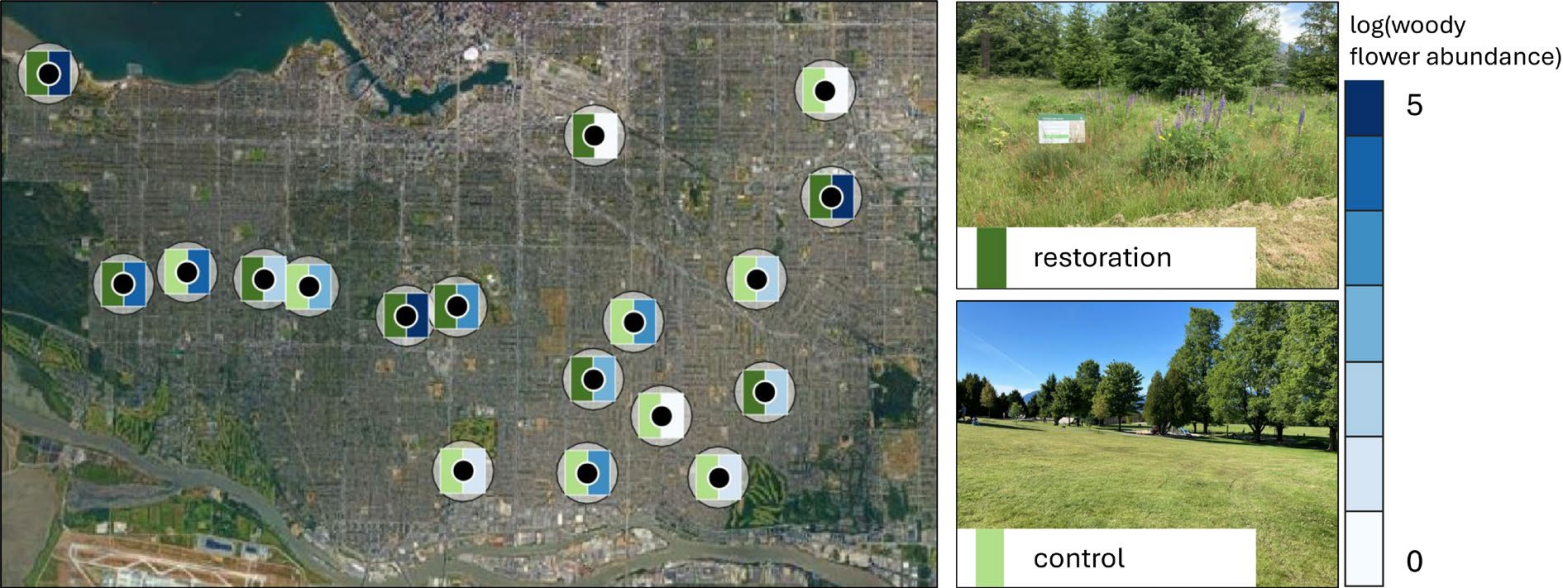
Site map. Map of the 18 study sites located in Vancouver, Canada. Left side rectangles drawn around each site marker indicate whether the site was restored (dark green) or served as a control (light green). Right side rectangles indicate the average log‐scale woody flower abundance measured across all surveys across all 3 years of the study, ranging from low (light blue) to high (dark blue). 500 m buffer circles illustrate that pollinator communities within each site are likely to be independent within years.

We surveyed plants and pollinators in nine restored parks and nine control parks over a period of 3 years (Figure 1). For the restored parks, a portion of turfgrass lawn (ranging from 0.37 to 6.52 ha, Figure S1) was left unmowed from April to August. Seven restorations were initiated in 2020; two others were established in previous years (Tables S1 and S2). These areas were seeded with a blend of grasses and wildflowers (Table S3). Control sites were mowed bi‐weekly throughout the summer. The climate of Vancouver is characterised by mild, dry summers and rainy winters (Meidinger and Pojar 1991). Management was conducted by the City of Vancouver.

In the first year of our study, we used high precision GPS to mark a site centroid that was surrounded by restored meadow (restored sites) or turfgrass lawn (control sites) for 50 m in all directions. We mapped a 1‐ha area square (100 m by 100 m) around each centroid, within which our plant and pollinator surveys were conducted.

To isolate the effects of restoration, we selected sites that shared similar landscape context, confirming that the proportion of impervious surface cover (mean = 61.0%, SD = 0.16; versus 69.4%, SD = 0.17) or tree canopy cover (mean = 19.8%, SD = 0.14; versus 24.1%, SD = 0.13) within a 500 m radius of each site did not differ for control versus restored sites (Figure S2). To ensure intra‐annual site independence, we selected sites that were separated by > 1 km distance (Figure 1), the estimated upper limit of typical wild bee foraging ranges (Greenleaf et al. 2007). Two pairs of sites were separated by < 1 km (Figure 1). Because we controlled landscape context through site selection, we did not include landscape variables in our analyses.

#### 2.1.2 Plant Surveys

We surveyed park plant and pollinator communities six times per year during the period of highest regional pollinator activity (May 1–August 15), in 2021, 2022 and 2023. Survey order was randomised. On each site visit, we surveyed herbaceous plant communities within the restoration or lawn, counting all floral units in twenty 1 m^2^ quadrats. Quadrats were placed every three meters along four transects radiating from the site centroid. A flower was considered a floral unit except for species with tightly packed inflorescences (e.g., Asteraceae and *Trifolium*), where each inflorescence was recorded as one floral unit (Guezen and Forrest 2021). We then counted all floral units on all woody plants occurring within the 1‐ha survey area. For trees with inaccessible branches, we estimated total floral units as the average per inflorescence multiplied by number of inflorescences (Kremen and Williams 2007).

#### 2.1.3 Pollinator Surveys

Plant surveys were performed on the same day as pollinator surveys, between 10 AM and 4 PM, when temperatures were above 16°C, average wind speeds below 2 m/s, and skies mostly‐sunny or full sun (Guezen and Forrest 2021). If weather did not permit a pollinator survey, we returned as soon as possible. We walked a spiral transect through the survey area, using a sweep‐net to capture all bees and hoverflies interacting with flowers. We netted for a total of 20 min on each survey, pausing the timer to handle captured insects. We limited surveying at any single patch of flowers to a maximum of 2 min to ensure sufficient site coverage. We deposited voucher specimens in the UBC Spencer Entomological Museum and our laboratory collection. Taxonomically unresolved or difficult groups were classified at the finest resolution possible (Table S4), and some readily identifiable species were released after surveys (Table S5). Although we captured honey bees ( *Apis mellifera* ), we did not include them in our analyses.

We inferred pollinator specialisation using the 5244 plant–pollinator interactions we observed, combined with 12,402 interactions from other studies from our region (Guzman, Kelly, and Elle 2023). We grouped plant species by genus and then calculated pollinator specialisation as Bluthgen's *d* (*d'*) (Blüthgen, Menzel, and Blüthgen 2006) using *bipartite* in R (Dormann, Gruber, and Fruend 2008). *d'* is a network statistic that characterises the number of plant taxa that each pollinator species interacts while also considering the availability of each plant (Blüthgen, Menzel, and Blüthgen 2006). *d'* was calculated using all interactions from across the entire network to obtain a single score per pollinator species (M'Gonigle et al. 2015). Availability was approximated as the total number of interactions received by each plant genus (Blüthgen, Menzel, and Blüthgen 2006). Specialisation metrics for each pollinator species are provided in Appendix S2.

#### 2.1.4 Pollen Limitation Study

To determine the effect of restoration on pollination, we quantified pollen limitation in six restored parks and five control parks (Table S2). For this experiment, we used a native, annual plant— *Clarkia amoena* (‘farewell to spring’; Onagraceae). *Clarkia* are facultatively outcrossing plants that produce many‐seeded capsules. Previous studies have demonstrated that pollen limitation in *Clarkia* can be driven by the pollinator environment (Moeller et al. 2012). Additionally, *Clarkia* did not already occur in the parks, allowing us to control population density (Moeller 2006). Seeds were obtained from Satinflower Nurseries (Victoria, BC). In March 2022, we broadcasted seed over a generic greenhouse potting mix in 70 two‐gallon pots. Plants were maintained in an outdoor common garden just outside of Vancouver, BC. We thinned pots to a density of seven plants per pot and, after flowering began in late June, moved six pots to each of our sites.

We chose four plants from each pot to use for the pollen limitation experiment. Following Moeller (2006), we tagged one flower on each plant to receive pollen supplementation and another paired flower (immediately above or below on the same inflorescence) as a control. The remaining plants in each pot served as pollen donors. We kept pots at our sites for 8 days, visiting each site four times to conduct pollen supplementation. Pollen supplementation was performed by moving pollen from the anthers of donor flowers to the reflexed stigmas of treatment flowers using a dissecting needle (Figure S3) (Moeller 2006). We then returned plants to a common garden, conducted pollinator observations and then harvested capsules as they ripened, counting seeds in the laboratory. Our observations confirmed that *C. amoena* interacts with a range of pollinator species, including wild bees and hoverflies (Table S6).

### 2.2 Statistical Analyses

#### 2.2.1 Multi‐Species Dynamic Occupancy Model

We used a multi‐species dynamic occupancy model to estimate initial occurrence, colonisation and persistence. This model structure explicitly estimates and accounts for imperfect detection (i.e., failure to detect a species despite its presence) which reduces overinflation of dynamic change (Dorazio et al. 2010). To fit this model, we converted data into binary detection/non‐detections (Figure S4).

Under this framework, we let Z represent the occurrence state (presence or absence) for species *i* at site *j* in year *k*. Occurrence is assumed to be the outcome of a Bernoulli trial: $Z_{i,j,k}\sim \text{Bernoulli}\left(\Psi_{i,j,k}\right)$. For the first year of the study, we treated $\Psi_{i,j,k}$ as equivalent to an initial occurrence probability: if *k* = 1, then $\Psi_{i,j,k}=\Psi 1_{i,j,k}$. To estimate the rates of persistence ($\varphi_{i,j,k}$) or colonisation ($\gamma_{i,j,k}$), we considered $\Psi_{i,j,k}$ in latter years to be the outcome of $\Psi_{i,j,k}$ in the previous year multiplied by the corresponding rate: if *k* > 1, then $\Psi_{i,j,k}=\varphi_{i,j,k}\times \Psi_{i,j,k-1}+\gamma_{i,j,k}\times \left(1-\Psi_{i,j,k-1}\right)$. With this formulation, our model heavily weighs the likelihood of persistence ($\varphi_{i,j,k}$) if a species was more likely to have occurred in the previous year ($\Psi_{i,j,k-1}$ approaching 1); alternatively, our model heavily weighs the likelihood of colonisation ($\gamma_{i,j,k}$) if a species was less likely to have occurred in the previous year ($\Psi_{i,j,k-1}$ approaching 0). We then modelled heterogeneity in the probability of initial occurrence, colonisation and persistence as:

(1) $$\begin{array}{c} \text{logit}\left(\Psi 1_{i,j,k}\right)=\Psi 1_{0}\left[i\right]+\Psi 1_{1}\times d^{'}\left[i\right]+\Psi 1_{2}\times \text{restoration}\left[j\right]+\Psi 1_{3}\times \text{woody flower abundance}\left[j,k\right]\\ +\Psi 1_{4}\times d^{'}\left[i\right]\times \text{restoration}\left[j\right]+\Psi 1_{5}\times d^{'}\left[i\right]\times \text{woody flower abundance}\left[j,k\right] \end{array}$$

(2) $$\begin{array}{c} \text{logit}\left(\gamma_{i,j,k}\right)=\gamma_{0}\left[i\right]+\gamma_{1}\times d^{'}\left[i\right]+\gamma_{2}\times \text{restoration}\left[j\right]+\gamma_{3}\times \text{woody flower abundance}\left[j,k\right]\\ +\gamma_{4}\times d^{'}\left[i\right]\times \text{restoration}\left[j\right]+\gamma_{5}\times d^{'}\left[i\right]\times \text{woody flower abundance}\left[j,k\right]+\gamma_{6}\left[k\right] \end{array}$$

(3) $$\begin{array}{c} \text{logit}\left(\varphi_{i,j,k}\right)=\varphi_{0}\left[i\right]+\varphi_{1}\times d^{'}\left[i\right]+\varphi_{2}\times \text{restoration}\left[j\right]+\varphi_{3}\times \text{woody flower abundance}\left[j,k-1\right]\\ +\varphi_{4}\times d^{'}\left[i\right]\times \text{restoration}\left[j\right]+\varphi_{5}\times d^{'}\left[i\right]\times \text{woody flower abundance}\left[j,k-1\right]+\varphi_{6}\left[k\right] \end{array}$$

In Equations ((1), (2), (3))–((1), (2), (3)), $\Psi 1_{0}$, $\varphi_{0}$ and $\gamma_{0}$ represent species‐specific intercepts drawn from normal distributions with mean and variance parameters estimated from the data, for example, where $\Psi 1_{0}\left[i\right]\sim \text{Normal}\left(\mu_{\Psi 1_{0}},\sigma_{\Psi 1_{0}}\right)$. The remaining parameters in each equation represent the effects of the corresponding covariates on initial occurrence, colonisation and persistence. Most of our study species have annual life cycles (Danforth, Minckley, and Neff 2019). Accordingly, our estimates for colonisation and persistence are generally informed by two complete population cycles for each species. We classified restoration as a binary factor and scored woody plants by taking the average log‐transformed woody plant floral abundance recorded across all surveys within a year at a site. Before calculating woody plant floral abundance, we filtered out species that were not visited by pollinators during our study.

To account for imperfect detection, we assumed that the occurrence state of each species at each site is fixed within each year. This assumption is justifiable because most of our study species have annual life cycles and fixed nest locations (Danforth, Minckley, and Neff 2019). We then assumed that detection, V, on survey *l* was the outcome of a Bernoulli trial conditional on both a detection rate, p, and the occurrence state, Z: $V_{i,j,k,l}\sim \text{Bernoulli}\left(Z_{i,j,k}\times p_{i,j,k,l}\right)$ (Dorazio et al. 2010). We modelled heterogeneity in detection rate as

(4) $$\begin{array}{c} \text{logit}\left(p_{i,j,k,l}\right)=p_{0}\left[i\right]+p_{1}\times \text{degree}\left[i\right]+p_{2}\left[i\right]\times \text{date}\left[j,k,l\right]+p_{3}\left[i\right]\times {\left(\text{date}\left[j,k,l\right]\right)}^{2}+p_{4}\\ \times \text{survey}\;\text{specific}\;\text{abundance}\;\text{abundance}\left[j,k,l\right]+p_{5}\left[k\right] \end{array}$$

In Equation (4), $p_{0}$ represents species–specific intercepts drawn from a normal distribution with mean and variance parameters estimated from the data: $p_{0}\left[i\right]\sim \text{Normal}\left(\mu_{p_{0}},\sigma_{p_{0}}\right)$. $p_{1}$ represents the effect of degree (the number of plants that a species interacts with) on detection, which we hypothesised would positively explain variation in detection rate. We obtained degree from the network we constructed to estimate *d'* (Dormann, Gruber, and Fruend 2008). By including date[*j*,*k*,*l*] and date[*j*,*k*,*l*]^2^ in the model, detection probability was allowed to vary around a species‐specific phenological peak ($p_{2}$) with a species‐specific phenological decay ($p_{3}$) (M'Gonigle et al. 2015). We assumed that $p_{2}$ and $p_{3}$ are drawn from community distributions: $p_{2/3}\left[i\right]\sim \text{Normal}\left(\mu_{p_{2/3}},\sigma_{p_{2/3}}\right)$. $p_{4}$ represents an effect of survey‐specific flower abundance, accounting for both woody plant and herbaceous restoration flower abundance. $p_{5}$ allows detection rate to vary across years.

We fit the model using a Bayesian approach, with models written in *Stan* (Stan Development Team 2023b), implemented in R with *rstan* (Stan Development Team 2023a). We employed best practices for model development and fitting by: testing model effectiveness with simulation; using weakly‐informative priors to discourage unrealistic parameter values (Table S7); confirming sufficient mixing of chains (Gelman‐Rubin R‐hat values < 1.05), minimal within‐chain autocorrelation (effective sample size/steps > 0.1) and no divergent transitions (Figures S5 and S6); and running our model for a length of 4000 HMC steps, discarding the first 2000 (Stan Development Team 2023b; Gelman, Hill, and Vehtari 2020). Covariates were *z*‐score standardised for model fitting. We assessed model fit using visual posterior predictive checks (Figure S7). For each parameter, our models generated a posterior distribution of values that are consistent with our data and priors. When the middle 95% of the posterior distribution (95% BCI) for a parameter was above or below zero, we concluded strong certainty in a positive or negative effect of the corresponding covariate (Gelman, Hill, and Vehtari 2020). We interpreted positive or negative 50% BCI's as marginal certainty (Gelman, Hill, and Vehtari 2020).

We synthesised our estimates for initial occurrence, colonisation and persistence to determine the effects of habitat restorations on species richness patterns, following Dorazio et al. (2010). Specifically, we (1) simulated occurrence for each species for a site with versus without a restoration; (2) summed the number of species predicted to occur at each site type; and (3) reiterated for each set of parameters in our posterior distribution. We repeated this procedure for a site with average versus one standard deviation above average woody plant floral abundance.

#### 2.2.2 Pollen Limitation Analysis

To quantify pollen limitation, we divided the number of seeds produced by each control flower by the number of seeds produced by the paired supplemented flower (Larson and Barrett 2000). We removed pairs if the control flower produced more than twice the seeds as the supplemented flower or if the supplemented flower did not fruit, resulting in a final sample of 175 pairs. The response was bimodal; control flowers tended to produce either ~0%–50% or ~100% of seeds relative to supplemented flowers (Figure S8). We interpreted the lower range as reproduction mainly through self‐fertilisation and the upper range as receipt of adequate pollen (Moeller 2006). As such, we classified pollen limitation as a binary outcome: < 50% of seeds relative to the paired flower = pollen limited; > 50% = not pollen limited. We then fit a Bayesian logistic regression model where pollen limitation (Y) at a site *j* was considered to be the outcome of a Bernoulli trial: $Y_{j}\sim \text{Bernoulli}\left(\theta_{j}\right)$. Where $\beta_{0}$ represents a random site‐specific intercept ($\beta_{0}\left[j\right]\sim \text{Normal}\left(\mu_{\beta_{0}},\sigma_{\beta_{0}}\right)$), and where $\beta_{1}$ represents the effect of restoration, we modelled the probability of pollen limitation ($\theta_{j}$) using a logit‐link as:

(5) $$\text{logit}\left(\theta_{j}\right)=\beta_{0}\left[j\right]+\beta_{1}\times \text{herbaceous enhancement}\left[j\right]$$

The pollen limitation model was fit with *Stan* as described above (Figures S9–S11).

## 3 Results

**FIGURE 2:**
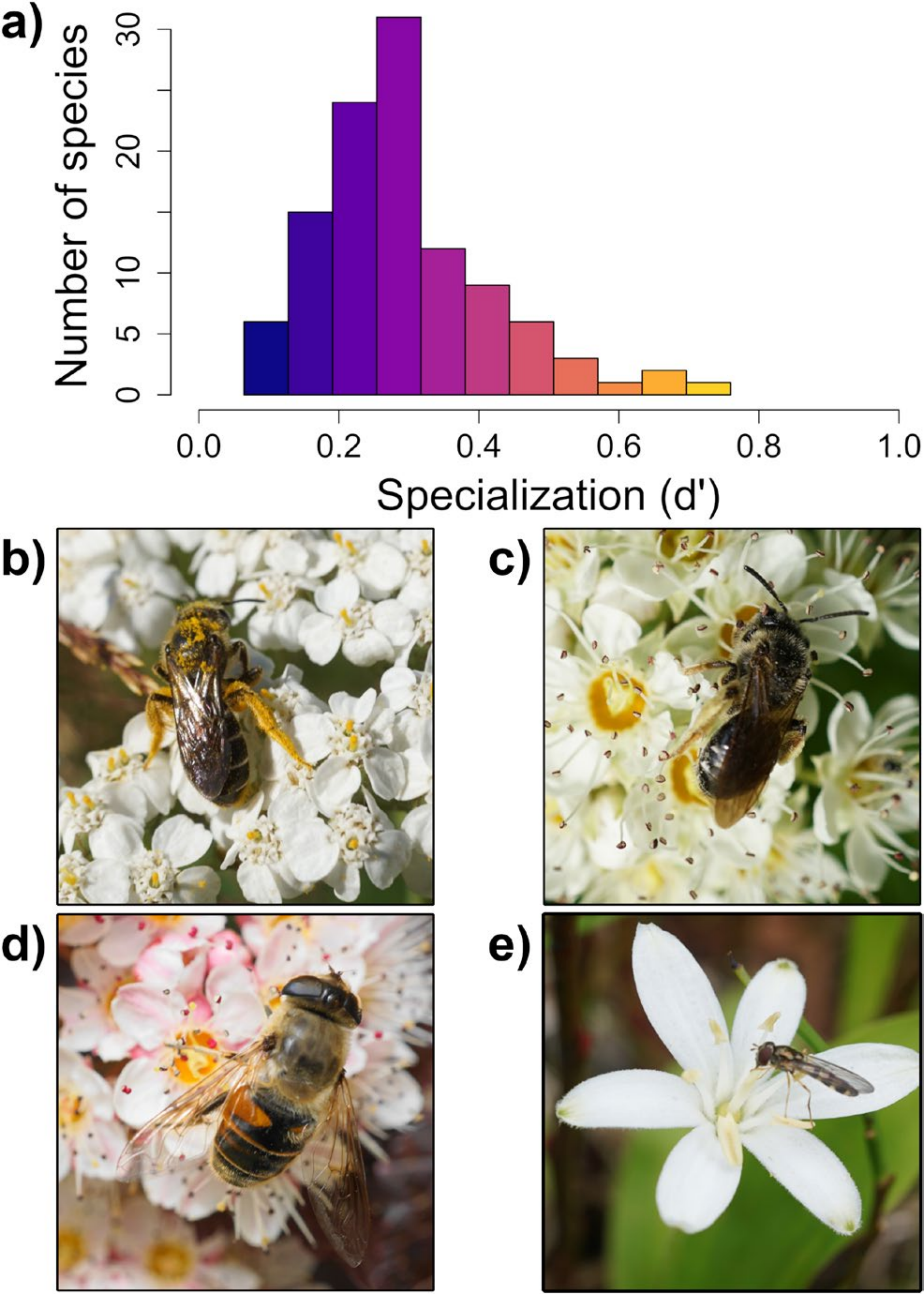
Species specialisation. We modelled the effects of species specialisation (*d'*) as a continuous predictor; however, we visualised the associations with restorations (Figure 3) and woody plant floral abundance (Figure 4) by predicting responses across 12 discrete specialisation bins coloured from purple (generalists; *d'* closer to 0) to yellow (specialists; *d'* closer to 1) (a). More generalised species included wild bees such as *Halictus rubicundus* (b) and several hoverflies from the drone fly group (*Eristalis* sps.) (d). More specialised species included wild bees such as *Andrena crataegi* (c) and hoverflies such as *Meligramma triangulifera* (e).

Restorations were associated with a marginal increase in flowering plant abundance and a clear increase in flowering plant richness (Figures S12–S15). Excluding plants that were not visited by pollinators, we recorded 59 forb species (Figure S16) and 18 woody plant species (Figure S17). We collected 5244 pollinators comprising 108 different species (Figure S18). The most abundant (Figure S16) and frequently visited (Figure S19) plants were non‐native herbaceous weeds such as clover, dandelion, yarrow and daisy. Mean pollinator *d'* was 0.29 (SD = 0.13) (Figure 2).

**FIGURE 3:**
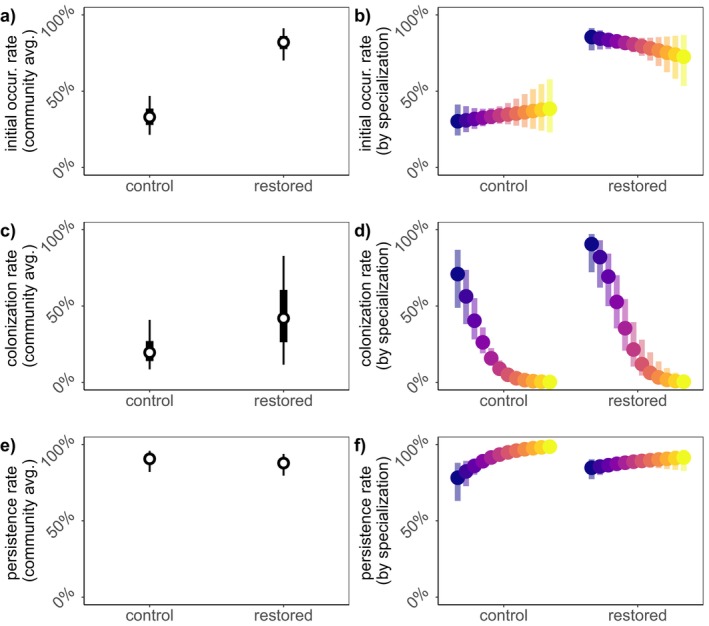
Effects of restorations on metacommunity dynamics—initial occurrence (a, b), colonisation (c, d) and persistence (e, f). Community mean responses to restorations are shown in the left column. 50% BCI's are illustrated in dark grey and 95% BCI's illustrated in light grey. Mean responses by specialisation bin (see Figure 2 for bin category values) are depicted in the right column, mapped from low specialisation (purple) to high specialisation (yellow) with 50% BCI's illustrated by transparent shades of corresponding colours. Responses to restorations are predicted for the mean level of woody plants observed in our study.

**FIGURE 4:**
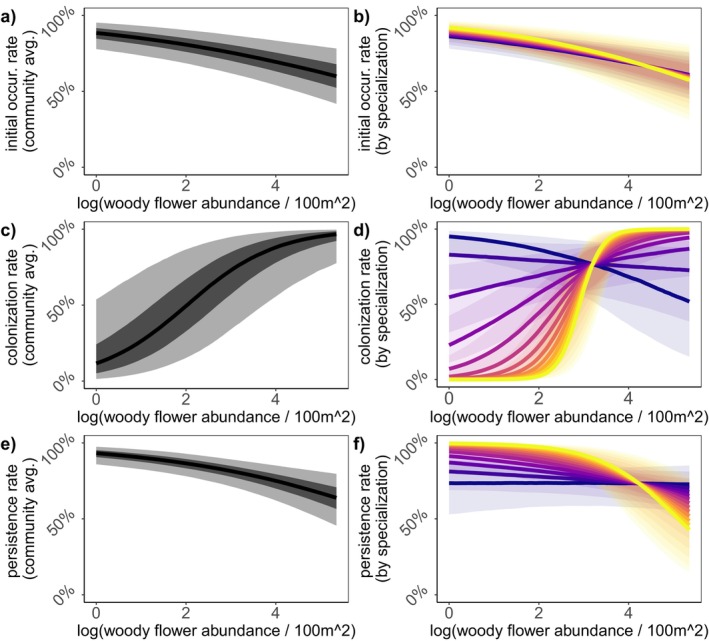
Effects of woody plant floral abundance on metacommunity dynamics—initial occurrence (a, b), colonisation (c, d) and persistence (e, f). Community mean responses to floral abundance from existing woody plants are shown in the left column. 50% BCI's are illustrated in dark grey and 95% BCI's illustrated in light grey. Mean responses by specialisation bin (see Figure 2 for bin category values) are depicted in the right column and mapped from low specialisation (purple) to high specialisation (yellow) with 50% BCI's illustrated by transparent shades of corresponding colours. Responses to woody plants are predicted for a site with restoration.

Restorations were positively associated with initial occurrence ($\Psi 1_{2}$ mean = 2.26, 95% BCI = [1.60, 3.13]) (Figure 3a), while woody plant floral abundance was negatively associated with initial occurrence ($\Psi 1_{3}$ mean = −0.48, 95% BCI = [−0.88, −0.20]) (Figure 4a). Specialisation was not associated with initial occurrence ($\Psi 1_{2}$ mean = 0.07, 95% BCI = [−0.56, 0.71]) (Figure 3b), nor did we find evidence for an interaction between specialisation and restoration ($\Psi 1_{4}$ mean = −0.21, 95% BCI = [−0.72, 0.40]) (Figure 3b) or woody plants ($\Psi 1_{5}$ mean = −0.04, 95% BCI = [−0.30, 0.22]) (Figure 4b).

We found marginal evidence for a positive association between restoration and colonisation rate ($\gamma_{2}$ mean = 1.02, 50% BCI = [0.44, 1.76]) (Figure 3c) while woody plants had a strong positive association ($\gamma_{3}$ mean = 1.60, 95% BCI = [0.69, 2.66]) (Figure 4c). Specialist species had lower colonisation rates than generalists ($\gamma_{1}$ mean = −1.32, 50% BCI = [−1.80, −0.84]) (Figure 3c). We found no interaction between restoration and specialisation on colonisation ($\gamma_{4}$ mean = −0.11, 95% BCI = [−1.49, 1.33]) (Figure 3d). In contrast, the positive effect of woody plant floral abundance on colonisation was stronger for specialists than for generalists ($\gamma_{5}$ mean = 1.36, 95% BCI = [0.26, 2.55]) (Figure 4d).

Restoration was not associated with persistence ($\varphi_{2}$ mean = −0.30, 95% BCI = [−1.34, 0.67]) (Figure 3e), and woody plants were negatively associated ($\varphi_{3}$ mean = −0.61, 95% BCI = [−1.04, −0.22]) (Figure 4e). Specialists tended to have higher persistence rates ($\varphi_{1}$ mean = 0.57, 50% BCI = [0.20, 0.93]) (Figure 3f). We found marginal evidence for small, negative interactions between specialisation and restoration ($\varphi_{4}$ mean = −0.42, 50% BCI = [−0.74, −0.08]) (Figure 3f) or woody plant abundance ($\varphi_{5}$ mean = −0.32, 50% BCI = [−0.49, −0.16]) (Figure 4f) on persistence.

Pollinators that interact with more plant species were easier to detect ($p_{1}$ mean = 0.98, 95% BCI = [0.74, 1.23]). After accounting for species‐specific phenology, survey‐specific floral abundance had a positive impact on detection ($p_{4}$ mean = 0.41, 95% BCI = [0.33, 0.50]) (Table S9).

We conducted an alternative a posteriori assessment of how specialisation mediates impacts of restoration and woody plants: We re‐fit our models with species‐specific random‐effects for each of our ecological predictors (rather than including effects of specialisation) and then assessed the correlation between *d'* and the estimated species‐specific slopes. *d'* was only a significant predictor of species‐specific effects of woody plants on colonisation rate (Figure S20), in agreement with our a priori test (Figures 3 and 4). With this approach, only two species had colonisation rates that were negatively associated with woody plant floral abundance (Figure S20).

**FIGURE 5:**
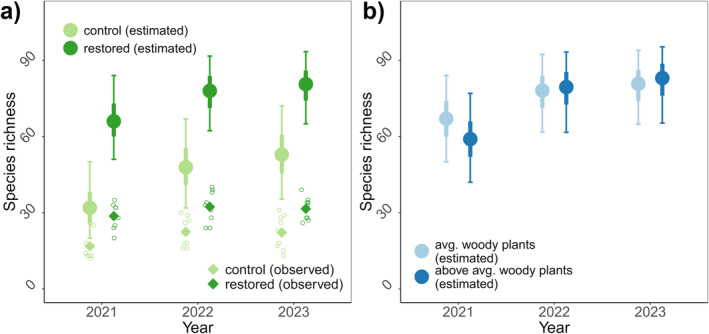
Consequences of metacommunity processes on biodiversity patterns. Given our estimates for species‐specific initial occurrence, colonisation and persistence, we estimated species richness for a control site (light green) and restored site (dark green) (a); and for a site with average woody plants (light blue) and with one standard deviation above average woody plants (dark blue) (standardised for having a restoration) (b). Filled circles indicate the posterior mean estimates; error bars indicate 50% and 95% BCI's. Our approach approximates species richness after accounting for imperfect detection of pollinator species. For (a), we also show the observed species richness for control and restored sites (site‐specific species richness indicated by open circles; mean species richness indicated by filled diamonds).

In 2021, pollinator species richness was higher in restored compared to control sites (mean increase in estimated species richness = 34 species; 95% BCI = [21, 47]) (Figure 5a). The difference in species richness endured across years (mean increase in estimated species richness in 2023 = 26 species; 95% BCI = [15, 39]) (Figure 5a). Estimated species richness (which accounts for imperfect detection) was approximately double our observed species richness (Figure 5a). Woody plants were not associated with differences in pollinator species richness (Figure 5b).

*Clarkia amoena* plants had a ~30% odds of pollen limitation in control parks ($\beta_{0}$ mean = −0.79, 95% BCI = [−1.51, −0.14]) (Figure S20). Restoration did not affect the probability of pollen limitation ($\beta_{1}$ mean = −0.05, 95% BCI = [−0.80, 0.69]) (Figure S21).

## 4 Discussion

Our study demonstrates that urban park restorations effectively promote long‐term biodiversity gains by rapidly assembling pollinator communities that are sustained. Specifically, we show that restoration had a strong, positive impact on the initial occurrence of pollinators, driving an immediate increase in species richness. The positive impact of restoration on the initial occurrence rate demonstrates that pollinator species from the wider regional pool track changes in their local environment, immediately dispersing to newly restored sites (Leibold et al. 2004). Moreover, we found that initial species richness gains are maintained in subsequent years due to a positive balance between colonisation and persistence. In contrast to the immediate impacts on pollinator communities that we observed, restorations in agricultural landscapes can require up to 5 or more years to achieve species richness gains (Blaauw and Isaacs 2014; M'Gonigle et al. 2015; Kremen, M'Gonigle, and Ponisio 2018). We hypothesise that the more heterogeneous availability of habitat in urban landscapes could offer more opportunity for pollinators to rapidly disperse to newly restored sites compared to conventional agricultural settings, consistent with theory that landscape moderates biodiversity patterns (Tscharntke et al. 2012).

The vast majority of studies examining the effect of restoration on pollinators have been performed in agricultural settings (Hyjazie and Sargent 2022). This is in spite of the fact that urban and agricultural landscapes are intrinsically different; the effects of restoration are unlikely to generalise from one to the other (Baldock et al. 2015; Theodorou 2022). Agricultural hedgerows, for example, are typically embedded in simplified landscapes dominated by crop monocultures and heavy pesticide usage (Tilman et al. 2001). Conversely, typical urban landscapes are a heterogenous patchwork of developed land and greenspace, with a high frequency of interconnected pollinator habitat in the form of parks, gardens and boulevard plantings with diverse floral resources (Baldock et al. 2019; Gerner and Sargent 2022). Many cities have also banned or restricted pesticide use (City of Vancouver 2018). The higher frequency of interspersed habitat in urban landscapes could enable faster colonisation by making it easier for species to disperse to restored sites (Driscoll et al. 2013; Fahrig 2017). Moreover, through a combination of these factors, urban landscapes could harbour a larger pool of species that are able to colonise restored sites (Baldock et al. 2015; Tscharntke et al. 2012). Clarifying how landscape differences combine with local restoration approaches to drive pollinator community assembly is critical for the design of biodiversity conservation initiatives that succeed over the long term.

The balance between colonisation and persistence that maintained species richness gains in our restored parks implies that biodiversity increases will be sustained in future years (Kery and Royle 2020). However, perturbations to the quality of the restorations could alter these long‐term trajectories (Kery and Royle 2020). For example, maturation of perennials or seeding of new plant species could further enhance the positive impacts of restoration (Kremen, M'Gonigle, and Ponisio 2018). Conversely, a shift in the plant community, for example, from insect‐pollinated forbs towards introduced grasses, could reverse biodiversity gains by negatively impacting persistence. To avoid this outcome, restorations should be continually managed to avoid dominance by introduced grasses (Luong et al. 2019).

Contrary to our predictions, existing woody plant floral abundance had a slight, negative association with initial occurrence and persistence of pollinators. This could be because our woody plants were typically trees. In landscape analyses, canopy cover can negatively correlate with pollinator biodiversity (Hanula, Horn, and O'Brien 2015; Hall et al. 2019), with many pollinator species preferring open, meadow‐like habitat (Ballare et al. 2019). Higher canopy cover at sites with more woody plants could have counteracted potential benefits from the floral resources they provide. Notably, woody plant abundance did not affect site pollinator species richness overall; the impacts on initial occurrence were marginal, and the negative effects on persistence were balanced by positive impacts on colonisation.

The positive impact of woody plant floral abundance on colonisation was stronger for dietary specialists than for generalists, suggesting that restored sites with more woody plants assemble communities with a higher number of specialists. Consistent with our results, woody plants in agricultural settings also had stronger effects on colonisation rates of specialists compared to generalists (M'Gonigle et al. 2015; Ponisio et al. 2019). While our restorations were initially seeded with wildflowers, they were ultimately dominated by spontaneous weeds from a narrow range of plant families (Asteraceae and Fabaceae families). Sites with woody plants, on the other hand, more often included a diversity of plant families absent from the applied seed mixes (e.g., Rosaceae, Caprifoliaceae, Cornaceae, Sapindaceae and Ericaceae). Many of our specialist pollinators interacted primarily with plants from these families, for example, *Andrena crataegi* (29/31 interactions with Rosaceae). Thus, sites with more woody plants integrated a diversity of plants that may have satisfied needs of species with stricter dietary preferences. Given the positive effect of woody plants on specialist colonisation rates and that the woody plants existed prior to the experiment, woody plants should have been associated with higher initial occurrence for specialists. We did not observe this. It is possible that restorations complemented the benefits of woody plants for specialists, for example, by supplying attractive nesting habitat or supplementary floral resources. These effects may have enabled more specialists to colonise sites with more woody plants after the start of the experiment. Further study is required to better understand how the existing plant community at a site interacts with the effects of restoration.

Despite the positive impacts of restoration on pollinator diversity, we did not detect an impact of restoration on pollen limitation of *Clarkia amoena* . We propose two possible explanations: First, the presence of common *C. amoena* pollinators in both restored and control sites (e.g., *Apis mellifera* and *Halictus spp*.) could have stabilised pollination rates from turnover in rare species (Kremen 2005; Genung et al. 2017). Alternatively, foraging activity of individual pollinators may shift depending on community composition, compensating for species losses (Kremen 2005). For example, in a study by Hallett et al. (2017), experimental exclusion of one pollinator species caused another to quadruple its visitation rate. We note that while restoration had no clear impact on pollen limitation, ~30% of experimental plants experienced pollen limitation. If pollen limitation in *C. amoena* is representative of other insect‐pollinated species in our region, their ability to set seed and persist could be compromised (Ashman et al. 2004).

Across the United States, turfgrass lawns cover three times more land surface area than any single irrigated crop (Milesi et al. 2005). In a study of North American urban landscapes, pollinator biodiversity was not associated with the area of turfgrass‐dominated greenspace, suggesting that they are not preferred pollinator habitat (Ulrich and Sargent 2024). Here, we demonstrate that restoring turfgrass can enhance pollinator biodiversity over the long term by encouraging the rapid dispersal of species that tend to persist. Applied across the expanse of lawns in urban landscapes, initiatives that encourage turfgrass restorations (through reduced mowing and added wildflower seeding) offer a promising strategy for mitigating pollinator declines (National Pollinator Garden Network 2019).

By examining the impacts of restoration on pollinator metacommunity dynamics in an urban landscape, we show that restored plant abundance and diversity interacts with pollinator traits to determine where pollinators occur. By creating a positive balance between the processes of colonisation and persistence, restoration is expected to sustain improved pollinator biodiversity over the long term. Importantly, our results indicate that the plant–pollinator community dynamics determined from studies in agricultural systems are not fully generalisable to urban landscapes. Future studies should work to reconcile the temporal differences in the effects of restoration in agricultural and urban settings to determine whether differences in landscape configuration, management and/or a higher availability of species drives the more rapid community assembly that we observed. Our findings have important implications for how other systems, including agroecosystems, should be designed to better support biodiversity.

## Author Contributions

J.U. and R.D.S. designed the study. J.U. collected the data, performed the analyses and wrote the first draft. R.D.S. supervised and both authors contributed to revisions.

### Peer Review

The peer review history for this article is available at https://www.webofscience.com/api/gateway/wos/peer‐review/10.1111/ele.70037.

## Supporting information


Data S1.

## Data Availability

Data are stored in a Dryad repository: https://doi.org/10.5061/dryad.t1g1jwtbp. Code required to conduct the analyses is stored in a linked Zenodo repository: https://zenodo.org/records/14188532.
