# Supplementary material for: Habitat Restorations in an Urban Landscape Rapidly Assemble Diverse Pollinator Communities That Persist
Source: Ecol Lett. 2024 Dec 31;28(1):e70037. doi: 10.1111/ele.70037 (PMC11687344; doi:10.1111/ele.70037)
Supplement: Supplementary file 1 — Data S1. [file ELE-28-0-s001.docx]

Supporting information to accompany “Habitat restorations in an urban landscape rapidly assemble diverse pollinator communities that persist”.

Jens Ulrich and Risa Sargent.

November, 2024.

***Appendix S1 –***

**Supplemental Tables**

**Table S1:** Site detail summary part 1.

**Table S2:** Site detail summary part 2.

**Table S3:** Wildflower seeding mix

**Table S4:** Taxonomic keys used for pollinator identification.

**Table S5:** List of pollinator species identified in the field.

**Table S6:** *Clarkia amoena* pollinator visitations.

**Table S7:** Prior distributions for occupancy model parameters.

**Table S8:** Prior distributions for pollen limitation model parameters.

**Table S9:** Posterior distributions for all parameters in the occupancy model.

**Supplemental Figures**

**Figure S1:** Map of all parks in Vancouver that were restored.

**Figure S2:** Landscape context for restored versus control sites.

**Figure S3:** *Clarkia amoena* hand pollination visuals.

**Figure S4:** Unique pollinator detections.

**Figure S5:** Traceplots for occupancy model.

**Figure S6:** Pairs plots for occupancy model.

**Figure S7:** Posterior predictive check for occupancy model.

**Figure S8:** Raw pollen limitation rates.

**Figure S9:** Traceplots for pollen limitation model.

**Figure S10:** Pairs plot for pollen limitation model.

**Figure S11:** Posterior predictive check for pollen limitation model.

**Figure S12:** Restoration plant abundance and diversity.

**Figure S13:** Effects of restoration on herbaceous plant abundance and richness.

**Figure S14:** Among-site variation in herbaceous floral resources.

**Figure S15:** Annual variation in herbaceous floral resources.

**Figure S16:** Herbaceous flower composition.

**Figure S17:** Woody flower composition.

**Figure S18:** Total unique pollinator detections by species.

**Figure S19:** Frequency of interactions by species.

**Figure S20:** *A posteriori* assessment pollinator specialization effects.

**Figure S21:** Effects of enhancements on pollen limitation.

***Appendix S2***

Appendix S2 is a large table (.xlsx format). Appendix S2 includes the complete list of species detected in the study, along with information on number of detections, specialization and phenology. Metadata are provided within the .xlsx file. The table can be accessed from our permanent Dryad repository: <https://doi.org/10.5061/dryad.t1g1jwtbp>.

**Table S1:** Site detail summary part 1.

| **Park Name** | **Restoration category** | **Woody plant flower abundance (average across 3 years; units in standard deviations above/below the mean)** | **Herbaceous enhancement initiation year** |
| --- | --- | --- | --- |
| balaclava | control | 0.509 | n/a |
| bobolink | control | -0.764 | n/a |
| china creek | restoration | -1.085 | pre-2020 |
| falaise | restoration | 2.182 | 2020 |
| gordon | control | -0.896 | n/a |
| kensington | control | 0.107 | n/a |
| killarney | restoration | -0.623 | 2020 |
| locarno | restoration | 1.559 | 2020 |
| memorial south | restoration | 0.041 | pre-2020 |
| moberly | control | 0.473 | n/a |
| oak meadows | restoration | 0.936 | 2020 |
| prince of wales | restoration | -0.73 | 2020 |
| queen elizabeth | restoration | 0.436 | 2020 |
| quilchena | control | -0.421 | n/a |
| rupert | control | -1.085 | n/a |
| slocan | control | -0.661 | n/a |
| west memorial | restoration | 0.817 | 2020 |
| winona | control | -0.794 | n/a |

**Table S2:** Site detail summary part 2.

| **Park Name** | **Percent impervious surface cover (500 m radius)** | **Percent tree cover (500 m radius)** | **Used for pollen limitation study?** | **Other comments** |
| --- | --- | --- | --- | --- |
| balaclava | 47% | 41% | yes |  |
| bobolink | 71% | 16% | yes |  |
| china creek | 75% | 18% | no |  |
| falaise | 84% | 8% | yes | Mowed prematurely in 2021 and 2023 |
| gordon | 81% | 9% | no |  |
| kensington | 83% | 10% | yes |  |
| killarney | 76% | 11% | yes |  |
| locarno | 46% | 34% | yes |  |
| memorial south | 74% | 15% | no |  |
| moberly | 88% | 6% | yes |  |
| oak meadows | 59% | 26% | yes |  |
| prince of wales | 49% | 32% | yes |  |
| queen elizabeth | 55% | 24% | no | Mowed prematurely in 2022 |
| quilchena | 40% | 47% | yes |  |
| rupert | 74% | 15% | no |  |
| slocan | 76% | 18% | no |  |
| west memorial | 40% | 49% | yes |  |
| winona | 66% | 15% | no |  |

**Table S3:** Wildflower seeding mix for herbaceous restorations.

| **Plant species** | **Nativity to British Columbia, Canada** |
| --- | --- |
| *Coreopsis lanceolata* – large leaved coreopsis | Non-native |
| *Gaillardia pulchella* - blanket flower | Non-native |
| *Centaurea cyanus* – bachelor button | Non-native |
| *Linum lewisii* – Lewis flax | Native |
| *Lupinus latifolius* – Arctic lupine | Native |
| *Lupinus polyphyllus* – large leaved lupine | Native |
| *Lupinus sericeus* – silky lupine | Native |
| *Symphotrichum douglassii* – Douglas aster | Native |
| *Trifolium incarnatum* – crimson clover | Non-native |
| *Trifolium pratense* – red clover | Non-native |
| *Plectritis congesta* – seablush | Native |
| **Grass seed:**  65% *Bromus carinatus* (California Brome),  2% *Deschampsia cespitosa* (Tufted Hairgrass),  15.5% Festuca idahoensis (Idaho Fescue),  12% *Koeleria macrantha* (Junegrass) | Native grass seed blend |

**Table S4:** Bees (families Andrenidae, Apidae, Colletidae, Halictidae and Megachilidae) and hoverflies (Syrphidae) were identified in the field or in the lab using a combination of reference specimens from the UBC Spencer Entomological Museum and the following keys. We could not confidently identify some pollinators to the species level due to unresolved taxonomy, no identification key for our region or otherwise uncertain morphology. These groups were classified at the finest taxonomic resolution possible as listed here.

| **Family** | **Genus** | **Taxonomic keys used for identification.** |
| --- | --- | --- |
| Andrenidae | *Andrena* | LaBerge, W.E., 1989. A revision of the bees of the genus Andrena of the Western Hemisphere. Transactions of the American Entomological Society, 115(1). |
| Apidae | *Bombus* | Williams, P.H., Thorp, R.W., Richardson, L.L. and Colla, S.R., 2014. Bumble bees of North America: an identification guide (Vol. 89). Princeton University Press. |
|  | *Ceratina* | Daly, H.V., 1973. Bees of the genus Ceratina in America north of Mexico (Hymenoptera: Apoidea). University of California Entomological Publications. Vol. 74, pp. 120-pp). |
|  | *Melissodes* | LaBerge, W.E., 1956. A revision of the bees of the genus Melissodes in North and Central America. Part I (Hymenoptera, Apidae). University of Kansas Science Bulletin, 37(18). |
|  | *Nomada* | No taxonomic key for this genus for our region. Grouped at the genus level. |
| Colletidae | *Colletes* | Identified using reference specimens |
|  | *Hylaeus* | Snelling, R.R., 1983. Studies on North American bees of the genus Hylaeus. Bulletin, Southern California Academy of Sciences, 82(1). |
| Halictidae | *Halictus* | Identified using reference specimens |
|  | *Lasioglossum* (Dialictus) | Gibbs, J., 2010. Revision of the metallic species of Lasioglossum (Dialictus) in Canada (Hymenoptera, Halictidae, Halictini). Zootaxa, 2591(1), pp.1-382. |
|  | *Lasioglossum* (Sphecodogastra) | No taxonomic key for this subgenus for our region. Grouped at the subgenus level. |
|  | *Lasioglossum (sensu stricto)* | McGinley, R.J., 1986. Studies of Halictinae (Apoidea: Halictidae), I: revision of new world Lasioglossum curtis. |
|  | *Sphecodes* | No taxonomic key for this genus for our region. Grouped at the genus level. |
| Megachilidae | *Anthidium* | Gonzalez, V.H. and Griswold, T.L., 2013. Wool carder bees of the genus Anthidium in the Western Hemisphere (Hymenoptera: Megachilidae): diversity, host plant associations, phylogeny, and biogeography. Zoological Journal of the Linnean Society, 168(2), pp.221-425. |
|  | *Coelioxys* | No taxonomic key for this genus for our region. Grouped at the genus level. |
|  | *Heriades* | Hurd, P.D. and Michener, C.D., 1955. The megachiline bees of California (Hymenoptera: Megachilidae). BULLETIN OF THE CALIFORNIA INSECT SURVEY, 3 |
|  | *Hoplitis* | Cooper, K.W., 1948. A Revision of the American Species of Hoplitis (Hymenoptera, Megachilidae). |
|  | *Megachile* | Sheffield, C.S., Ratti, C., Packer, L. and Griswold, T., 2011. Leafcutter and mason bees of the genus Megachile Latreille (Hymenoptera: Megachilidae) in Canada and Alaska. Canadian Journal of Arthropod Identification, 18(1), pp.1-107. |
|  | *Osmia* | We identified four species with the draft key and by comparison with the museum reference specimens. We identified an fifth morphospecies. We were unable to match males to our females and consequently filtered them out of our records. |
| Syrphidae | *Eristalis*, *Eristalinus, Fazia, Helophilus, Merodon, Myathropa, Spilomyia, Syritta* | Dankowicz, Z. and Dankowicz, E.. Field/Photo Id for Flies. https://sites.google.com/view/flyguide |
|  | *Eupeodes, Lapposyrphus, Meligramma, Melanostoma, Parasyrphus, Platycheirus, Scaeva, Sphaerophoria, Syrphus, Toxomerus* | J.R. Vockeroth. 1992. The insects and archnids of Canada, Part 18: The flower flies of the subfamily Syrphinae of Canada, Alaska and Greenland. Agriculture Canada. |
|  | *Eumerus* | No taxonomic key for this genus for our region. Grouped at the genus level. |

**Table S5:** Some common, readily recognizable species were identified at the end of a survey round (after chilling on ice during the survey round) and released back to the sites. This was intended to reduce sampling impacts and to reduce interference with a simultaneous mark-recapture study conducted at the sites which focused on some of these species. If we were uncertain of the ID, we kept the specimen as a voucher. We kept at least one voucher per year for each of these species to confirm ID’s. We generally still kept males for all of these species which tend to be harder to identify in the field.

| Species name | Notes |
| --- | --- |
| *Apis mellifera* | Honey bees were common and distinctive. |
| *Bombus vosnesenskii* | These bumble bees generally readily identified by clear and distinctive colour banding patterns. Note that *Bombus caligonosus* is not differentiable from *B. vosnesenskii* in the field. There could be overlap with our ID’s for *B. vosnesenskii* containing some *B.* c*aligonosus*, however, the latter species is deemed to be rare in our region. |
| *Bombus mixtus* | These bumble bees generally readily identified by clear and distinctive colour banding patterns. |
| *Bombus flavifrons* | These bumble bees generally readily identified by clear and distinctive colour banding patterns. |
| *Bombus melanopygus* | These bumble bees generally readily identified by clear and distinctive colour banding patterns. |
| *Bombus sitkensis* | These bumble bees generally readily identified by clear and distinctive colour banding patterns. |
| *Bombus nevadensis* | These bumble bees generally readily identified by clear and distinctive colour banding patterns. |
| *Halictus rubicundus* | Only *Halictus* with non-metallic integument and lacking prominent facial spine in our region. |
| *Agapostemon texanus* | Only *Agapostemon* with green abdomen in our region. |
| *Anthidium oblongatum* | Orange tegula (compare with *A. manicatum*). |
| *Anthidium manicatum* | Black and yellow tegula (compare with *A. oblongatum*). |
| *Melissodes microstictus* | No other *Melissodes* in this size range in our region. |

**Table S6:** *Clarkia amoena* pollinator interactions. With a team of 3 observers, we recorded pollinator interactions with open flowers in a common garden in a residential neighborhood in Vancouver. Observations were conducted within the days immediately following the pollen limitation experiment. We considered a pollinator to be interacting with the flower if it touched the reproductive organs (anthers and/or stigma) of one or more flowers. We did not double count individuals that touched more than one flower during a foraging bout, however, if a pollinator left the common garden array and then returned during the observation interval it may have been double counted. Order Diptera refers to true flies while order Hymenoptera includes bees and other wasps.

| Date | Observation length (minutes) | Order | (finest taxonomic resolution possible) |
| --- | --- | --- | --- |
| 7.7.2022 | 45 | Diptera | Syrphidae |
|  |  | Diptera | Syrphidae |
|  |  | Diptera | Syrphidae |
|  |  | Diptera | Syrphidae |
|  |  | Diptera | Syrphidae |
|  |  | Diptera | Syrphidae |
|  |  | Diptera | Syrphidae |
|  |  | Diptera | Syrphidae |
|  |  | Diptera | Syrphidae |
|  |  | Diptera | Syrphidae |
|  |  | Diptera | Syrphidae |
|  |  | Diptera | Diptera - Other |
|  |  | Diptera | Diptera - Other |
|  |  | Diptera | Diptera - Other |
|  |  | Diptera | Diptera - Other |
|  |  | Hymenoptera | Halictus sp. |
|  |  | Hymenoptera | Halictus sp. |
|  |  | Hymenoptera | Halictus sp. |
|  |  | Hymenoptera | Halictus sp. |
|  |  | Hymenoptera | Halictus sp. |
|  |  | Hymenoptera | Halictus sp. |
|  |  | Hymenoptera | Halictus sp. |
|  |  | Hymenoptera | Ceratina sp. |
|  |  | Hymenoptera | Ceratina sp. |
|  |  | Hymenoptera | Nomada sp. |
| 7.8.2022 | 15 | Diptera | Syrphidae |
|  |  | Diptera | Syrphidae |
|  |  | Diptera | Diptera - Other |
|  |  | Apidae | Ceratina sp. |
| 7.9.2022 | 15 | Diptera | Syrphidae |
|  |  | Diptera | Syrphidae |
|  |  | Diptera | Syrphidae |
| 7.11.2022 | 30 | Diptera | Syrphidae |
|  |  | Diptera | Syrphidae |
|  |  | Diptera | Syrphidae |
|  |  | Diptera | Diptera - Other |
|  |  | Diptera | Diptera - Other |
|  |  | Diptera | Diptera - Other |
|  |  | Hymenoptera | Apis mellifera |
|  |  | Hymenoptera | Apis mellifera |
|  |  | Hymenoptera | Megachile sp. |

**Table S7:** Prior distributions for occupancy model parameters. In the context of logistic regression, a centered prior clusters the prior probability distribution around no effect of the parameter on probability of the outcome with, if all other parameters are set to 0, a 50% odds of success.

| **Parameter** | **Interpretation** | **Prior Distribution** | **Justification** |
| --- | --- | --- | --- |
| **Detection parameters** | | | |
| $\mu_{p_{0}}$ | Global intercept | Normal(0,2) | Centered weak prior |
| $\sigma_{p_{0}}$ | [Community variance] intercept | Normal(0,2) | Centered weak prior |
| $p_{1}$ | Effect of increasing diet breadth | Normal(0,2) | Centered weak prior |
| $\mu_{p_{2}}$ | [Community mean] effect of day of year | Normal(0,2) | Centered weak prior |
| $\sigma_{p_{2}}$ | [Community variance] effect of day of year | Normal(0,2) | Centered weak prior |
| $\mu_{p_{3}}$ | [Community mean] effect of day of year squared | Normal(0,2) | Centered weak prior |
| $\sigma_{p_{3}}$ | [Community variance] effect of day of year squared | Normal(0,2) | Centered weak prior |
| $p_{4}$ | Effect of increasing survey-specific flower abundance | Normal(0,2) | Centered weak prior |
| $p_{5}$ | Effect of year | Normal(0,0.25) | Strong centered prior limiting the effects of annual variation on detection probability |
| **Initial occurrence parameters** | | | |
| $\mu_{{\Psi1}_{0}}$ | Global intercept | Normal(0,2) | Centered weak prior |
| $\sigma_{{\Psi1}_{0}}$ | [Community variance] intercept | Normal(0,2) | Centered weak prior |
| ${\Psi1}_{1}$ | Effect of increasing diet specialization | Normal(0,2) | Centered weak prior |
| ${\Psi1}_{2}$ | Effect of restoration | Normal(0,2) | Centered weak prior |
| ${\Psi1}_{3}$ | Effect of woody plants | Normal(0,2) | Centered weak prior |
| ${\Psi1}_{4}$ | Interaction between diet specialization and restoration | Normal(0,1) | Centered prior tightened to keep interactions closer to zero without sufficient evidence from the data |
| ${\Psi1}_{5}$ | Interaction between diet specialization and woody plants | Normal(0,1) | Centered prior tightened to keep interactions closer to zero without sufficient evidence from the data |
| **Colonization parameters** | | | |
| $\mu_{\gamma_{0}}$ | Global intercept | Normal(0,1) | Centered weak prior |
| $\sigma_{\gamma_{0}}$ | [Community variance] intercept | Normal(0,1) | Centered weak prior |
| $\gamma_{1}$ | Effect of increasing diet specialization | Normal(0,2) | Centered weak prior |
| $\gamma_{2}$ | Effect of restoration | Normal(0,2) | Centered weak prior |
| $\gamma_{3}$ | Effect of woody plants | Normal(0,2) | Centered weak prior |
| $\gamma_{4}$ | Interaction between diet specialization and restoration | Normal(0,1) | Centered weak prior |
| $\gamma_{5}$ | Interaction between diet specialization and woody plants | Normal(0,1) | Centered weak prior |
| $\gamma_{6}$ | Effect of year | Normal(0,0.25) | Strong centered prior limiting the effects of annual variation on colonization probability |
| **Persistence parameters** | | | |
| $\mu_{\varphi_{0}}$ | Global intercept | Normal(0,1) | Centered weak prior |
| $\sigma_{\varphi_{0}}$ | [Community variance] intercept | Normal(0,1) | Centered weak prior |
| $\varphi_{1}$ | Effect of increasing diet specialization | Normal(0,2) | Centered weak prior |
| $\varphi_{2}$ | Effect of restoration | Normal(0,2) | Centered weak prior |
| $\varphi_{3}$ | Effect of woody plants | Normal(0,2) | Centered weak prior |
| $\varphi_{4}$ | Interaction between diet specialization and restoration | Normal(0,1) | Centered weak prior |
| $\varphi_{5}$ | Interaction between diet specialization and woody plants | Normal(0,1) | Centered weak prior |
| $\varphi_{6}$ | Effect of year | Normal(0,0.25) | Strong centered prior limiting the effects of annual variation on persistence probability |

**Table S8:** Prior distributions for pollen limitation model parameters. In the context of logistic regression, a centered prior clusters the prior probability distribution around no effect of the parameter on probability of the outcome with, if all other parameters are set to 0, a 50% odds of success.

| **Parameter** | **Interpretation** | **Prior Distribution** | **Justification** |
| --- | --- | --- | --- |
| $\mu_{\beta_{0}}$ | Global intercept | Normal(0,2) | Centered weak prior |
| $\sigma_{\beta_{0}}$ | [(Among-site) community variance] intercept | Normal(0,2) | Centered weak prior |
| $\beta_{1}$ | Effect of restoration | Normal(0,2) | Centered weak prior |

**Table S9:** Posterior distributions for all parameters. Continuous variables were z-score scaled; as such, estimates indicate the association between the response variable and an increase in 1 sd of the covariate. For categorical variables, estimates indicate the association between a change in the category and the response. Values are presented on the logit scale.

| **Parameter** | **Interpretation** | **Mean** | **Lower 95%** | **Lower 50%** | **Upper 50%** | **Upper 95%** |
| --- | --- | --- | --- | --- | --- | --- |
| **Detection parameters** | | | | | | |
| $\mu_{p_{0}}$ | Global intercept | -2.836 | -3.248 | -2.979 | -2.696 | -2.436 |
| $\sigma_{p_{0}}$ | [Community variance] intercept | 1.175 | 0.961 | 1.097 | 1.257 | 1.431 |
| $p_{1}$ | Effect of increasing diet breadth (degree) | 0.975 | 0.735 | 0.892 | 1.058 | 1.227 |
| $\mu_{p_{2}}$ | [Community mean] effect of julian date | 0.231 | -0.065 | 0.133 | 0.331 | 0.524 |
| $\sigma_{p_{2}}$ | [Community variance] effect of julian date | 1.369 | 1.138 | 1.286 | 1.460 | 1.651 |
| $\mu_{p_{3}}$ | [Community mean] effect of julian date squared | -0.750 | -0.929 | -0.806 | -0.694 | -0.595 |
| $\sigma_{p_{3}}$ | [Community variance] effect of julian date squared | 0.545 | 0.411 | 0.497 | 0.597 | 0.713 |
| $p_{4}$ | Effect of increasing survey-specific flower abundance | 0.412 | 0.331 | 0.383 | 0.441 | 0.496 |
| $p_{5}$ [2021] | Annual effect on detection | -0.21 | -0.51 | -0.31 | -0.10 | 0.09 |
| $p_{5}$ [2022] | Annual effect on detection | -0.01 | -0.30 | -0.11 | 0.09 | 0.27 |
| $p_{5}$ [2023] | Annual effect on detection | 0.18 | -0.11 | 0.08 | 0.28 | 0.47 |
| **Initial occurrence parameters** | | | | | | |
| $\mu_{{\Psi1}_{0}}$ | Global intercept | -0.705 | -1.412 | -0.950 | -0.464 | -0.013 |
| $\sigma_{{\Psi1}_{0}}$ | [Community variance] intercept | 2.058 | 1.483 | 1.838 | 2.299 | 2.862 |
| ${\Psi1}_{1}$ | Effect of restoration | 2.228 | 1.565 | 1.988 | 2.502 | 3.103 |
| ${\Psi1}_{2}$ | Effect of woody plant floral abundance | -0.477 | -0.797 | -0.586 | -0.375 | -0.177 |
| ${\Psi1}_{3}$ | Effect of specialization (*d’*) | 0.071 | -0.557 | -0.147 | 0.282 | 0.716 |
| ${\Psi1}_{4}$ | Interaction between restoration and specialization | -0.221 | -0.716 | -0.389 | -0.038 | 0.396 |
| ${\Psi1}_{5}$ | Interaction between woody plants and specialization | -0.040 | -0.289 | -0.120 | 0.039 | 0.217 |
| **Colonization parameters** | | | | | | |
| $\mu_{\gamma_{0}}$ | Global intercept | -1.417 | -2.587 | -1.820 | -0.992 | -0.172 |
| $\sigma_{\gamma_{0}}$ | [Community variance] intercept | 2.700 | 1.762 | 2.347 | 3.093 | 3.890 |
| $\gamma_{1}$ | Effect of restoration | 1.090 | -0.853 | 0.435 | 1.761 | 3.229 |
| $\gamma_{2}$ | Effect of woody plant floral abundance | 1.570 | 0.691 | 1.254 | 1.921 | 2.654 |
| $\gamma_{3}$ | Effect of specialization (*d’*) | -1.300 | -2.931 | -1.804 | -0.835 | 0.221 |
| $\gamma_{4}$ | Interaction between restoration and specialization | -0.118 | -1.497 | -0.569 | 0.337 | 1.334 |
| $\gamma_{5}$ | Interaction between woody plants and specialization | 1.351 | 0.258 | 0.963 | 1.747 | 2.549 |
| $\gamma_{6}$ [2021/2022] | Annual effect on colonization | -0.02 | -0.51 | -0.18 | 0.14 | 0.46 |
| $\gamma_{6}$ [2022/2023] | Annual effect on colonization | -0.06 | -0.53 | -0.22 | 0.09 | 0.40 |
| **Persistence parameters** | | | | | | |
| $\mu_{\varphi_{0}}$ | Global intercept | 2.265 | 1.390 | 1.955 | 2.608 | 3.333 |
| $\sigma_{\varphi_{0}}$ | [Community variance] intercept | 0.599 | 0.033 | 0.344 | 0.846 | 1.326 |
| $\varphi_{1}$ | Effect of restoration | -0.280 | -1.339 | -0.624 | 0.041 | 0.672 |
| $\varphi_{2}$ | Effect of woody plant floral abundance | -0.605 | -1.046 | -0.741 | -0.469 | -0.224 |
| $\varphi_{3}$ | Effect of specialization (*d’*) | 0.540 | -0.466 | 0.196 | 0.929 | 1.685 |
| $\varphi_{4}$ | Interaction between restoration and specialization | -0.397 | -1.444 | -0.735 | -0.082 | 0.501 |
| $\varphi_{5}$ | Interaction between woody plants and specialization | -0.315 | -0.871 | -0.485 | -0.156 | 0.107 |
| $\varphi_{6}$ [2021/2022] | Annual effect on persistence | -0.24 | -0.54 | -0.09 | 0.07 | 0.37 |
| $\varphi_{6}$ [2022/2023] | Annual effect on persistence | 0.23 | -0.20 | 0.08 | 0.39 | 0.66 |

**a)**
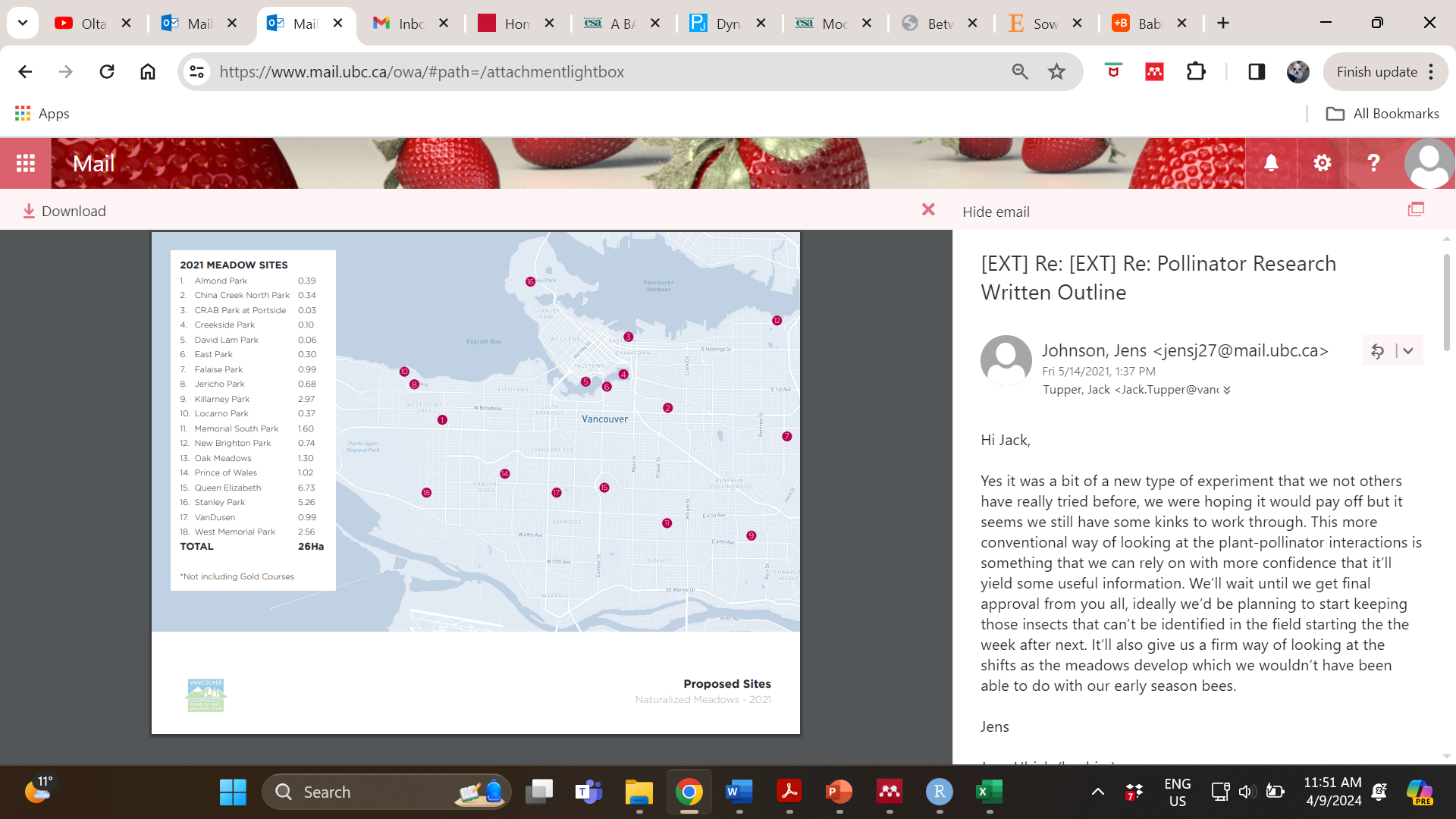


**b)**
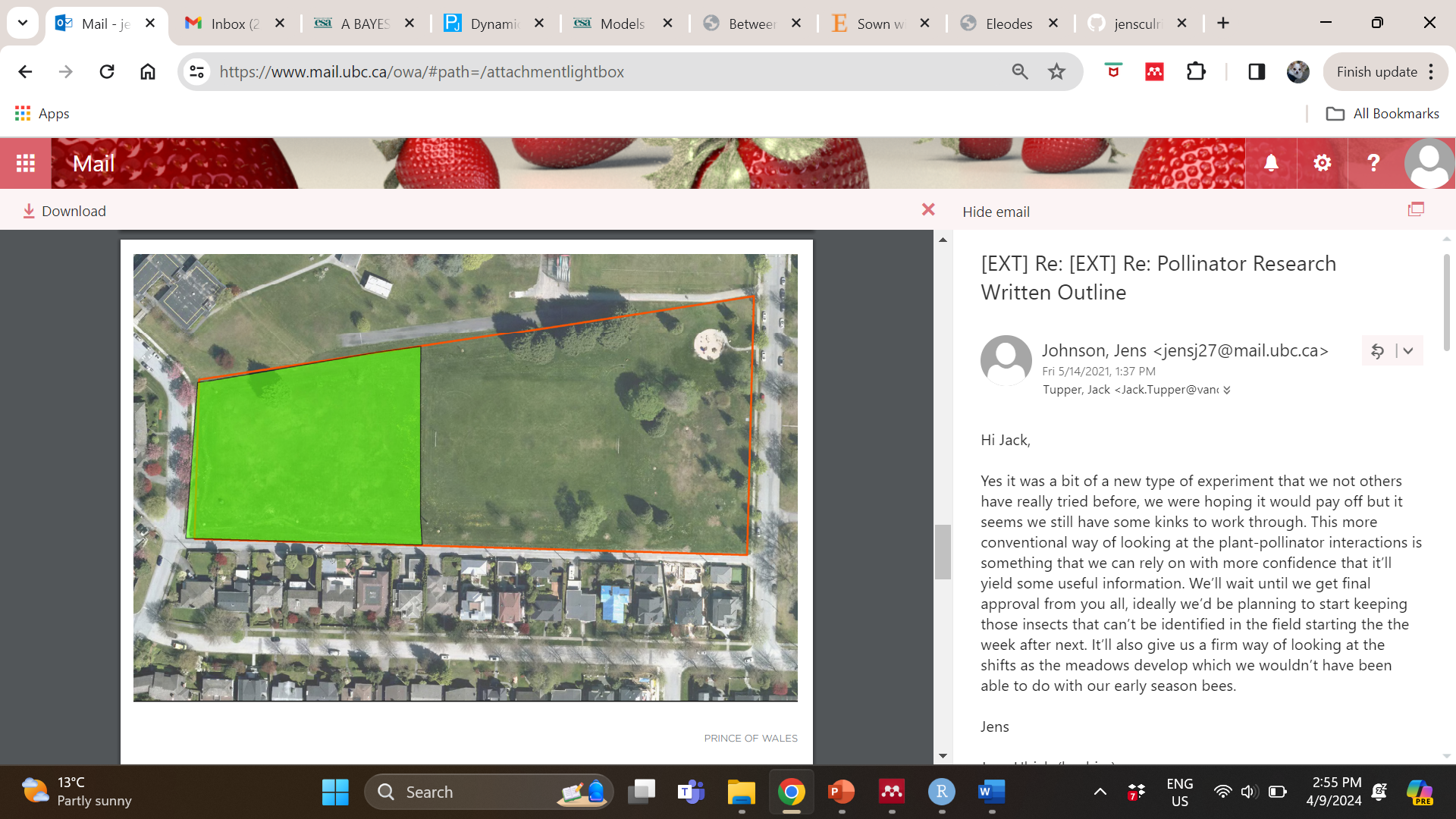


**Figure S1:** All parks in the city of Vancouver receiving restoration treatment. 18 Vancouver parks were restored (a). We selected 9 of these based on size and location to monitor and compared these against 9 paired control parks that did not receive a restoration. We did not survey all 18 restoration sites because some sites were too close to others by spatial distance (non-independent samples), the sites in the downtown core were simultaneously smaller and surrounded by high degree of impervious surface, and available time prohibited effective sampling at a larger number of sites. The restorations were installed in a portion of the park (not the entire park). An example for one of our study sites, Prince of Wales park (1.02 hectares of restoration) is shown as an example (b).

**a)**
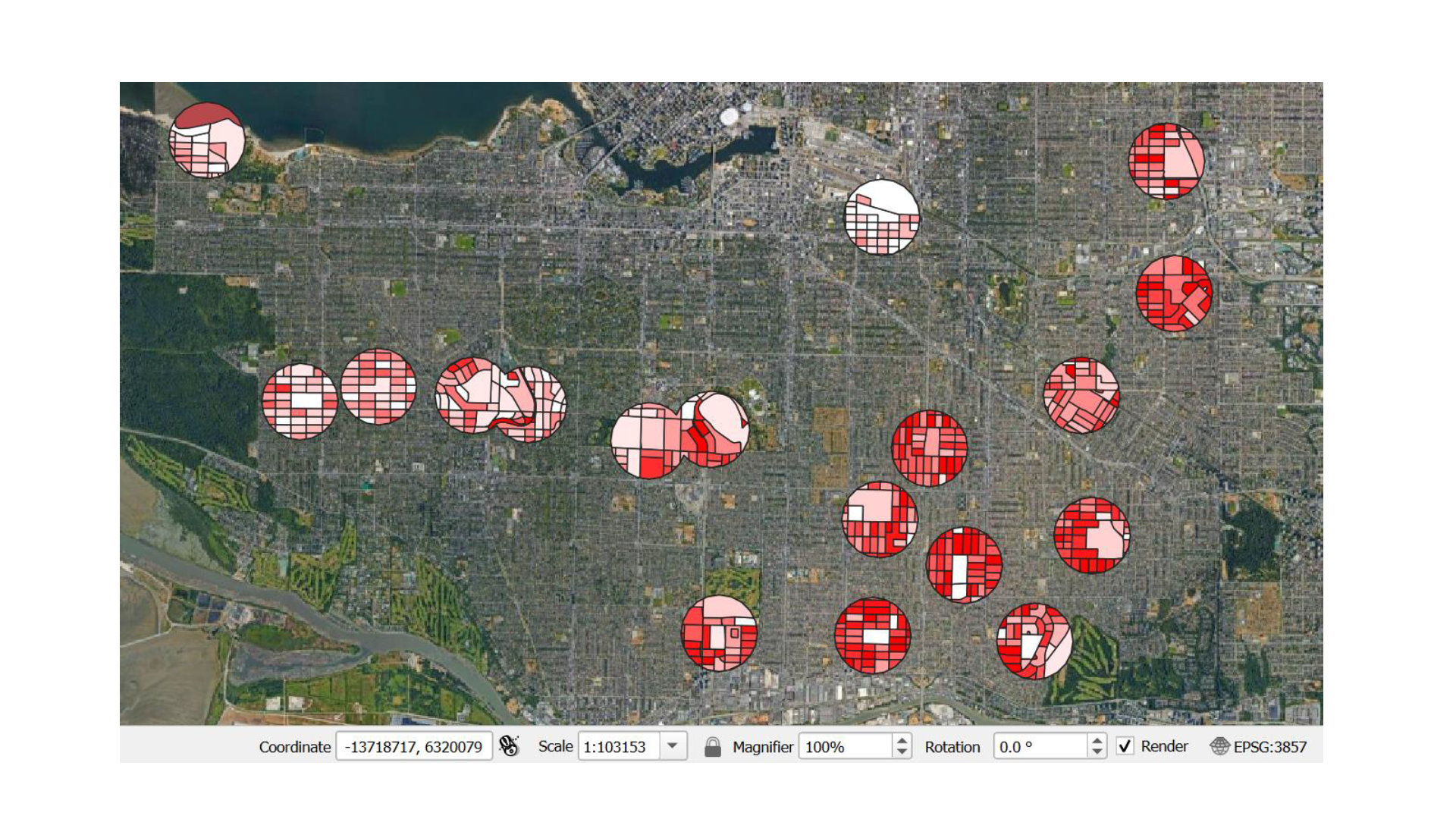


**b)**
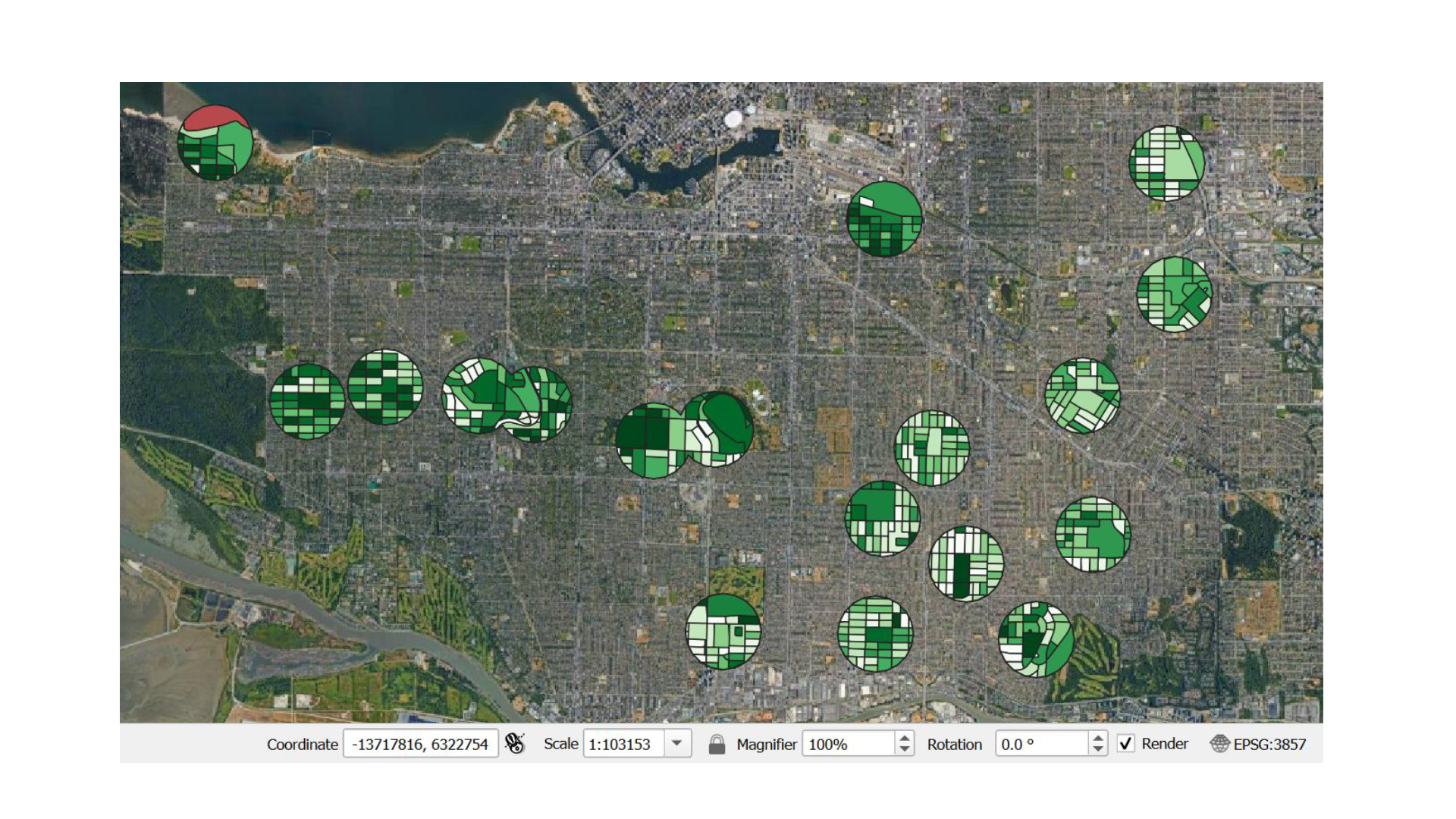


**c)**
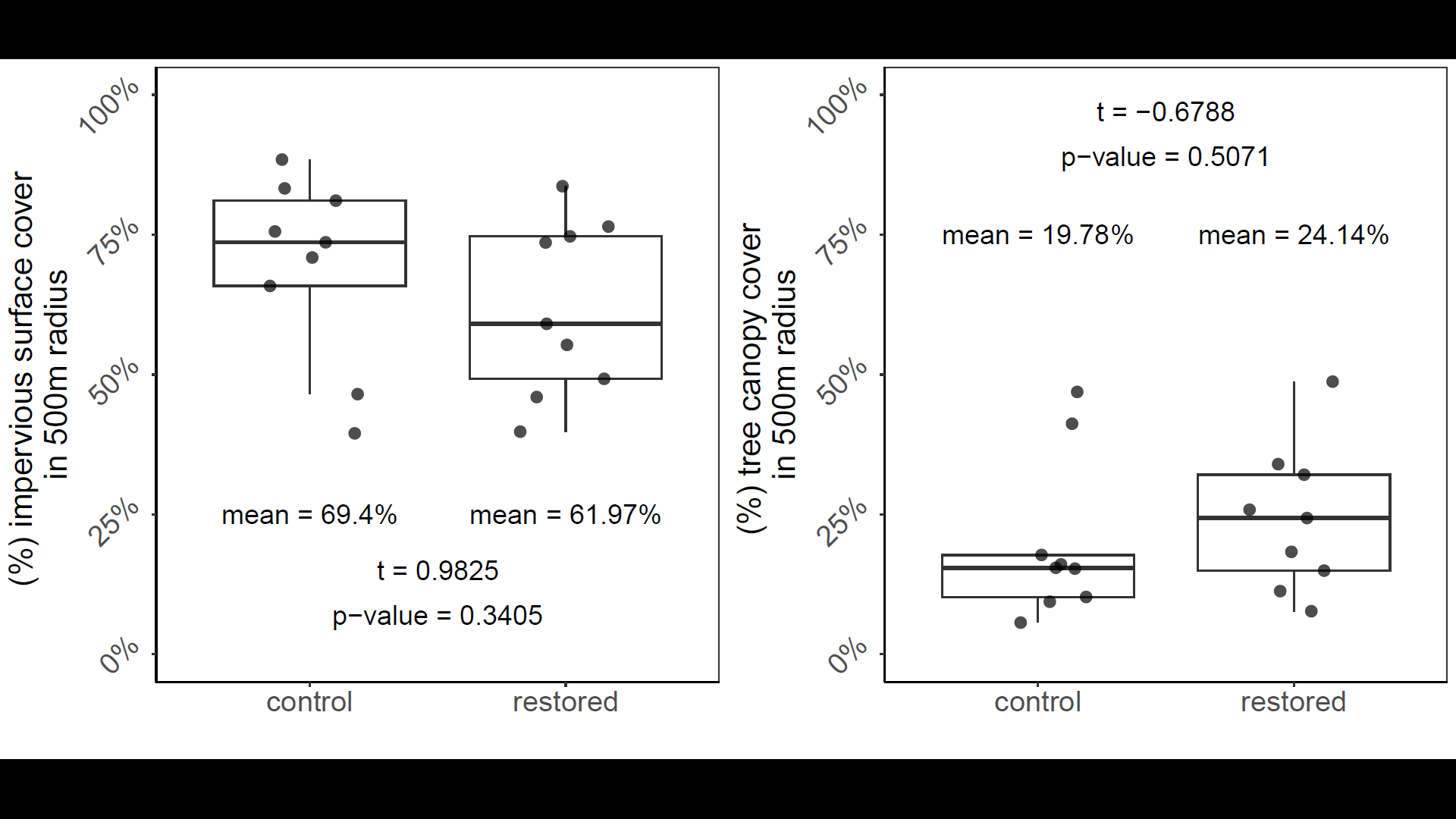


**Figure S2:** Landscape context. We calculated the percent impervious surface cover and the percent tree canopy cover within a 500 m radius of control sites versus restored sites. Cover was determined by clipping census block impervious surface (a) and tree canopy cover (b) layers to a 500 m radius buffer around each site, multiplying the census block cover percentage by the area of the census block contained within each landscape clip, summing the product for all census blocks within each site buffer, and then dividing the sum by the total area of the 500 m radius buffer. We compared the landscape context between control and restored sites using a two-sample t-test and found that there is no difference on average between the two site types, confirming that treatment is randomized with respect to landscape context (c). Mean impervious control = 69.4 (sd = 0.17) versus mean impervious restored = 61.0 (sd = 0.16); mean canopy control = 19.8 (sd = 0.14) versus mean canopy restored = 24.1 (sd = 0.13). Data were obtained from the Metro Vancouver Open Data Portal: <https://open-data-portal-metrovancouver.hub.arcgis.com/datasets/5a505c71327b4e73a6760058791d4258_6/explore?location=49.238743%2C-123.103738%2C13.25>


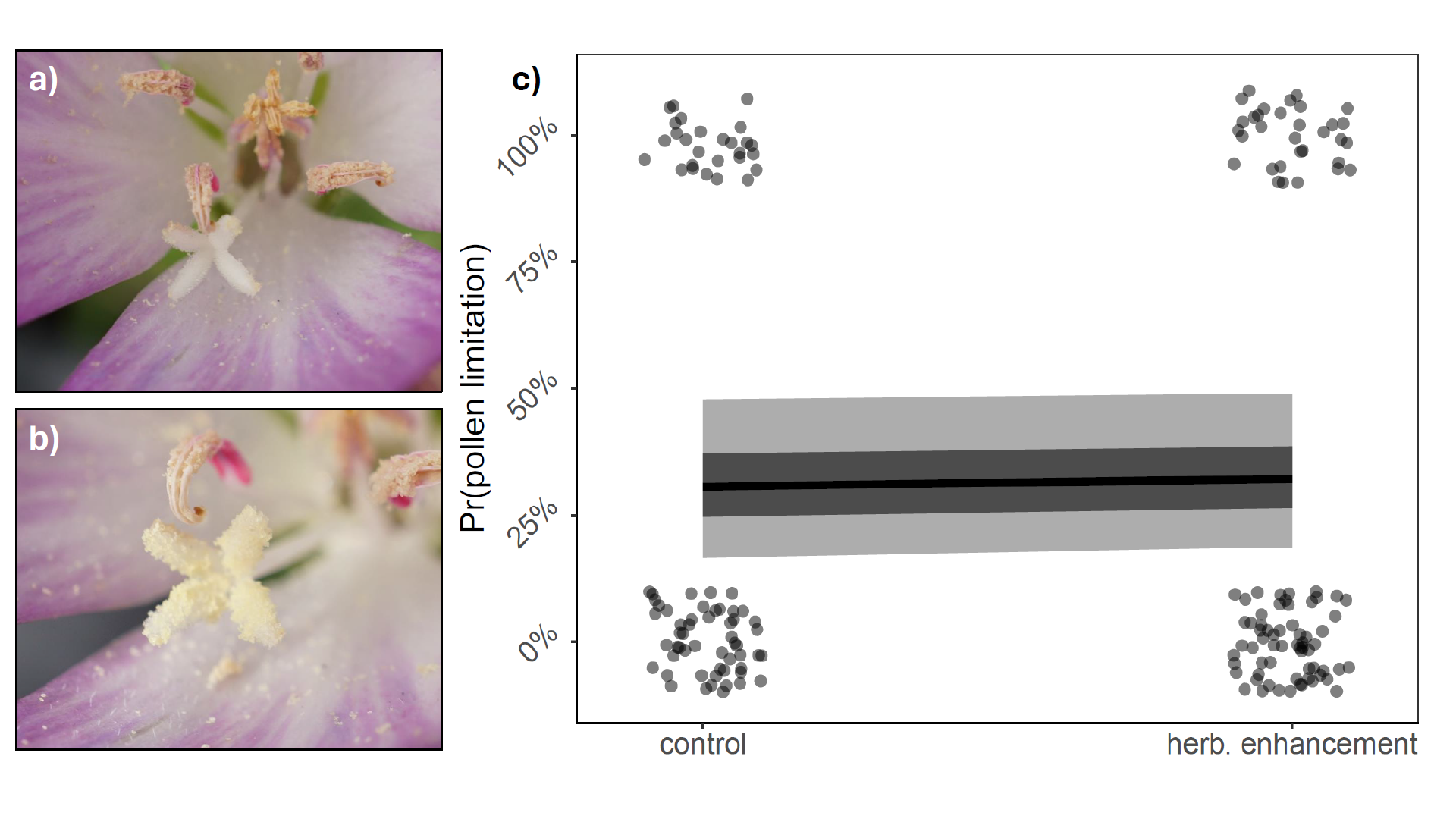


**Figure S3:** When stigmatic surfaces were receptive (a), we supplemented one flower per *Clarkia amoena* plant with a fresh load of conspecific pollen (b).

1.
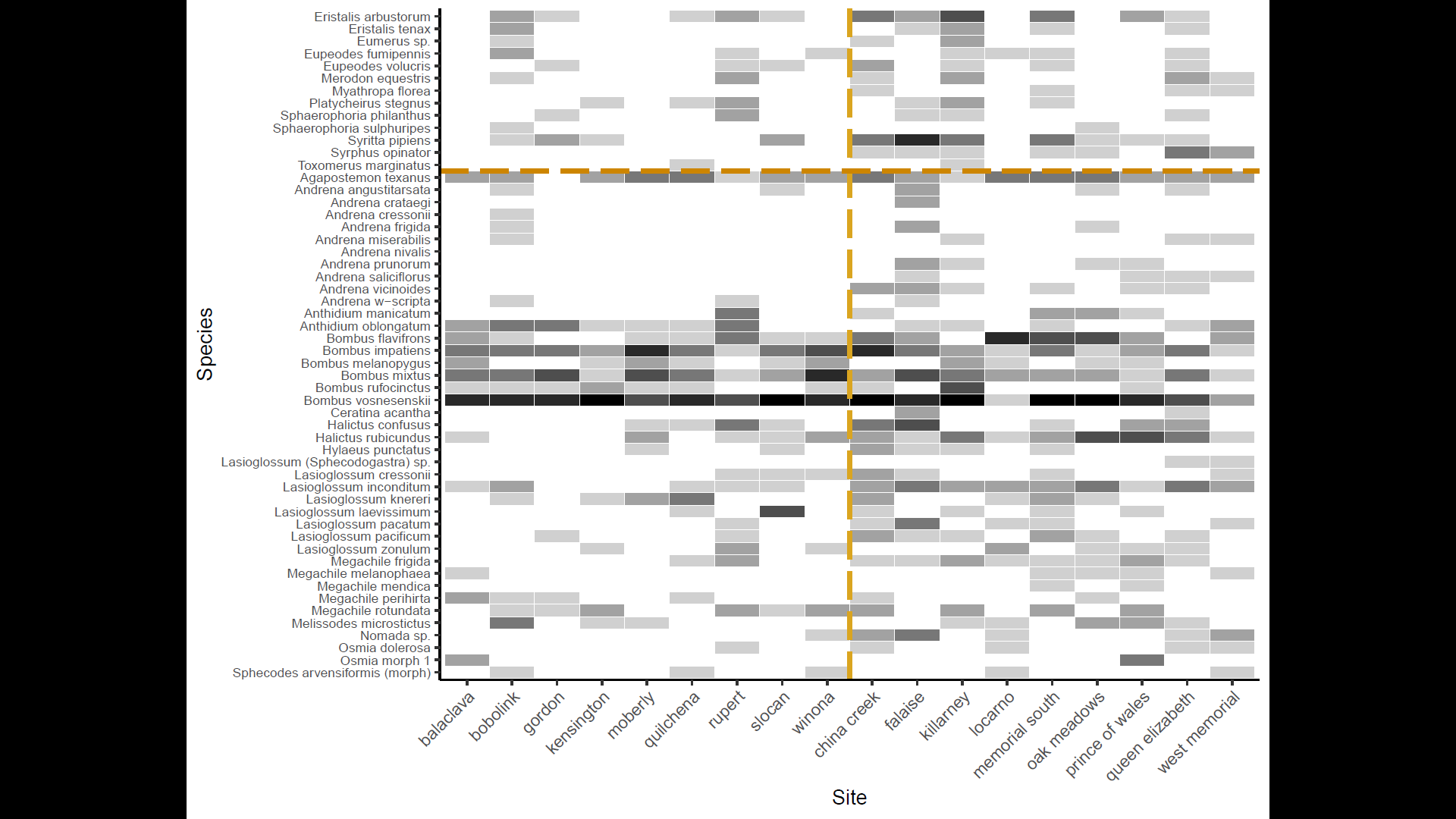

2.
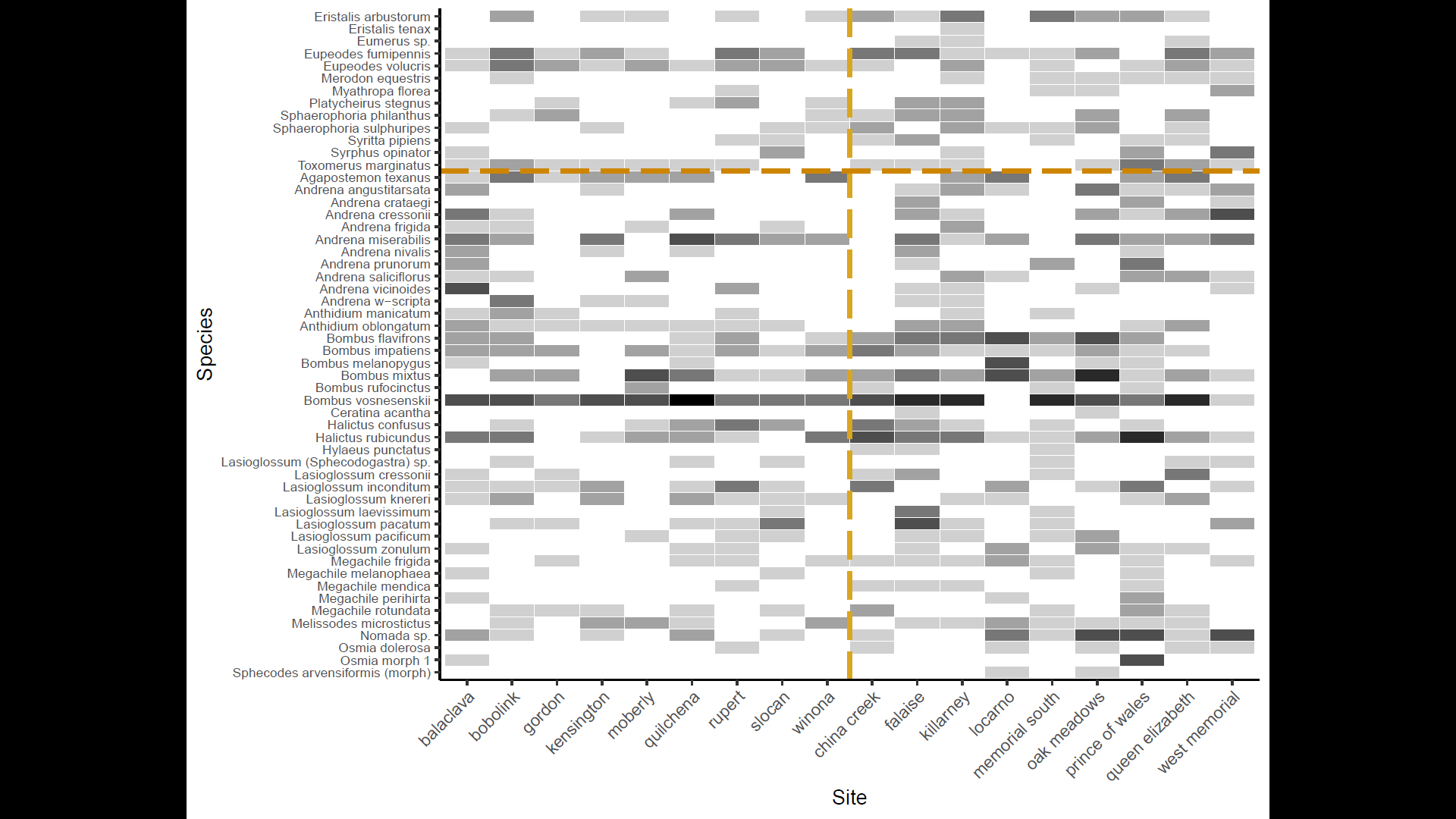

3.
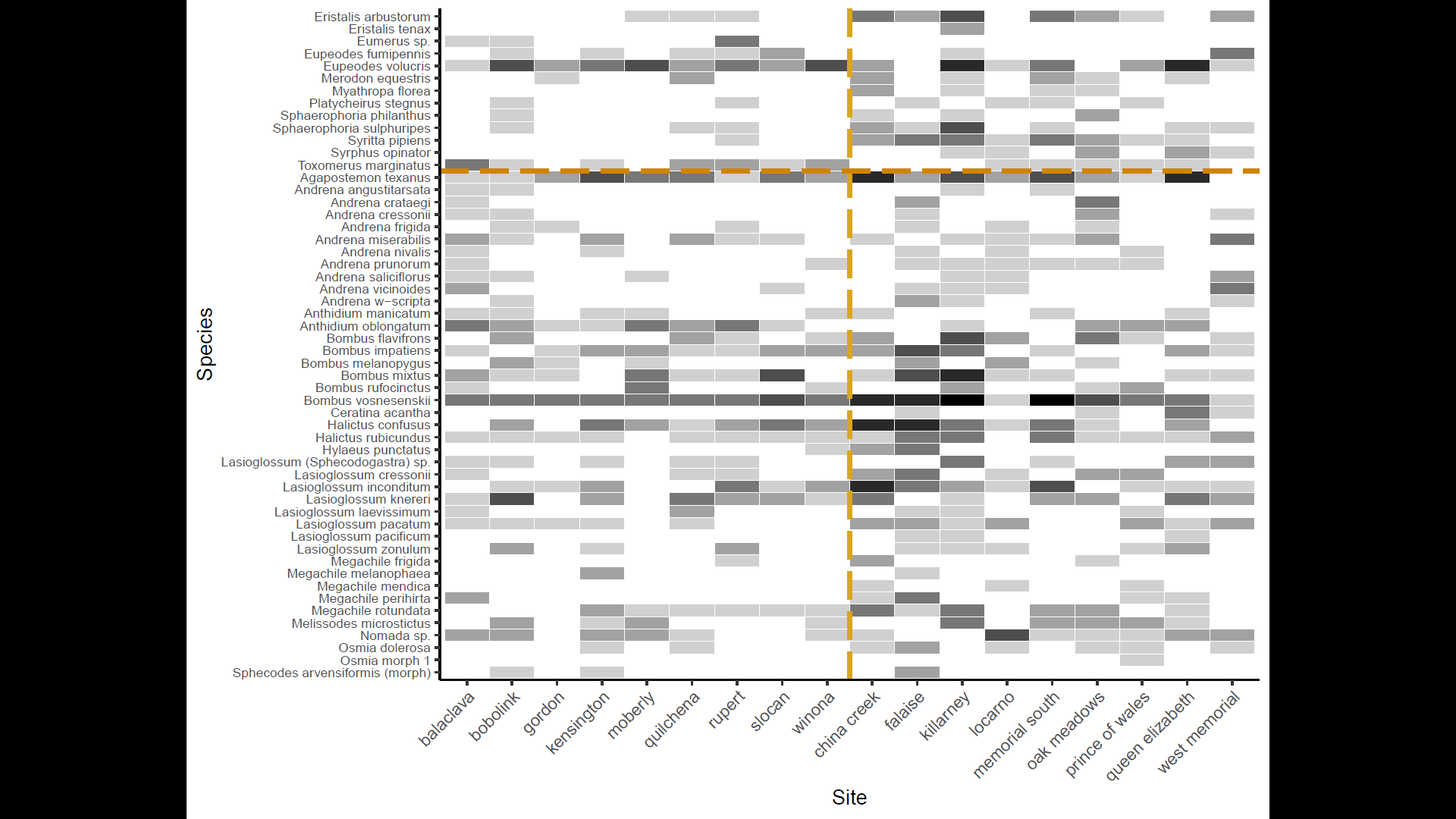


**Figure S4:** Unique pollinator detections. To fit an occupancy model to our data we converted abundance into binary detection/non-detection for each species at each site on each visit. Binary detection/non-detections are shown for a sample of species with hoverflies above the orange dashed line and wild bees below. Restored sites are displayed to the right of the yellow dashed line. The intensity of shading indicates the number of visits with a detection in each year ranging from 0 (white) to 1 – 5 (light to dark grey) to 6 (black). Binary detections are shown for 2021 (a), 2022 (b) and 2023 (c). For legibility, only species detected 10 or more times are shown here.

1.
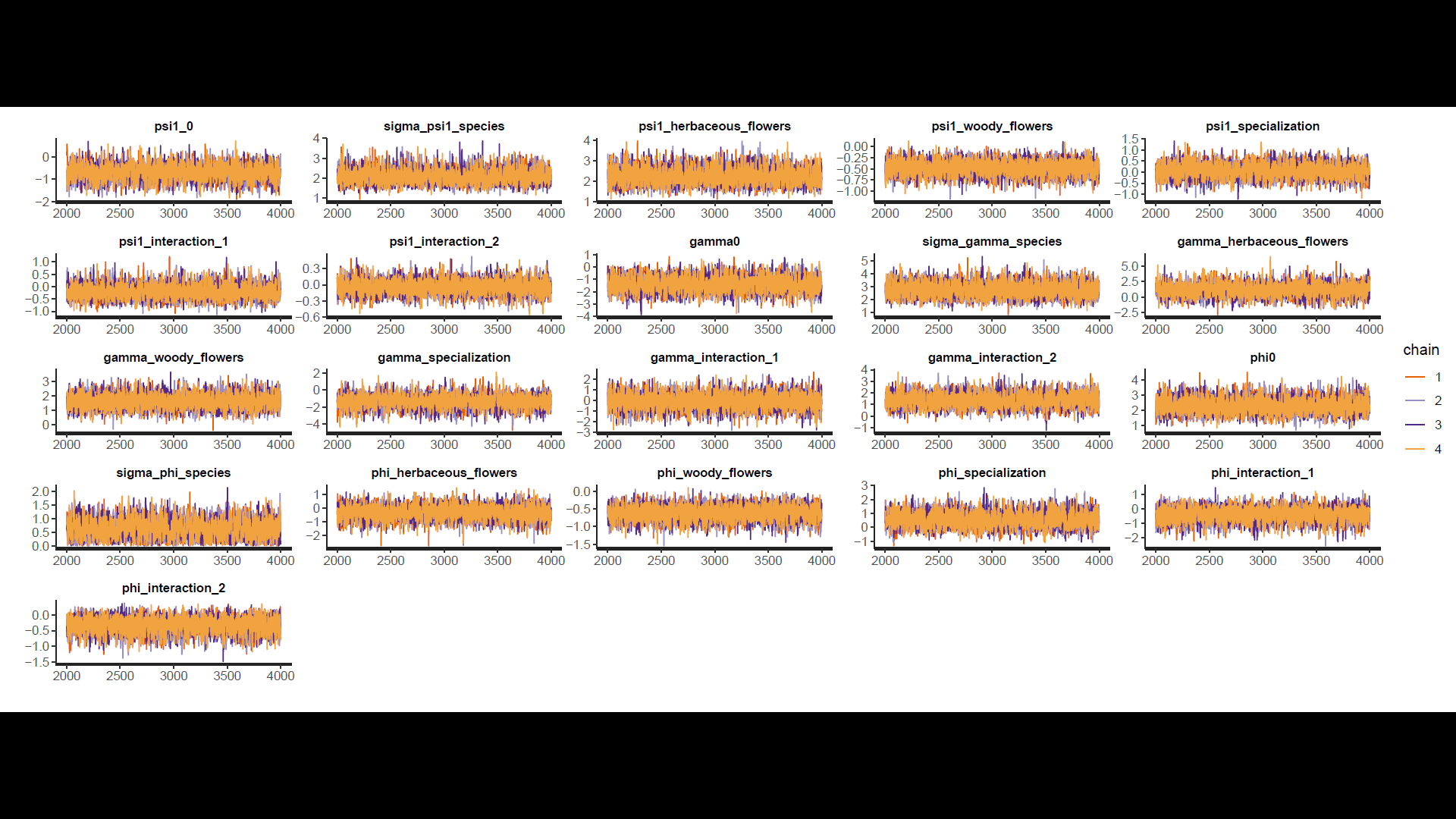

2.
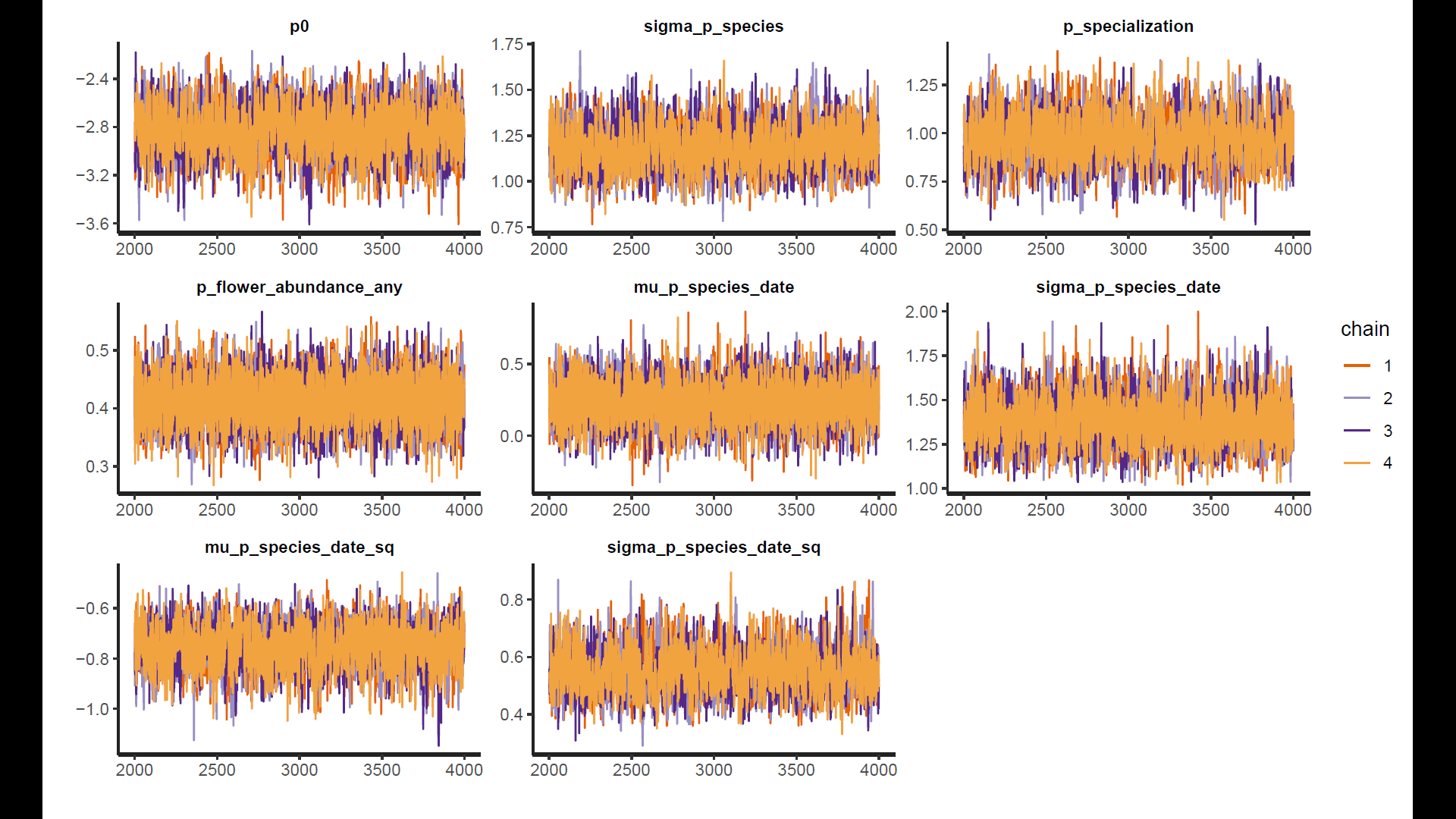


**Figure S5:** Traceplots and pairs plots for occupancy model. Overlapping HMC traces for ecological parameters (a) and detection parameters (b) indicate that all 4 chains have converged on a similar parameter space.


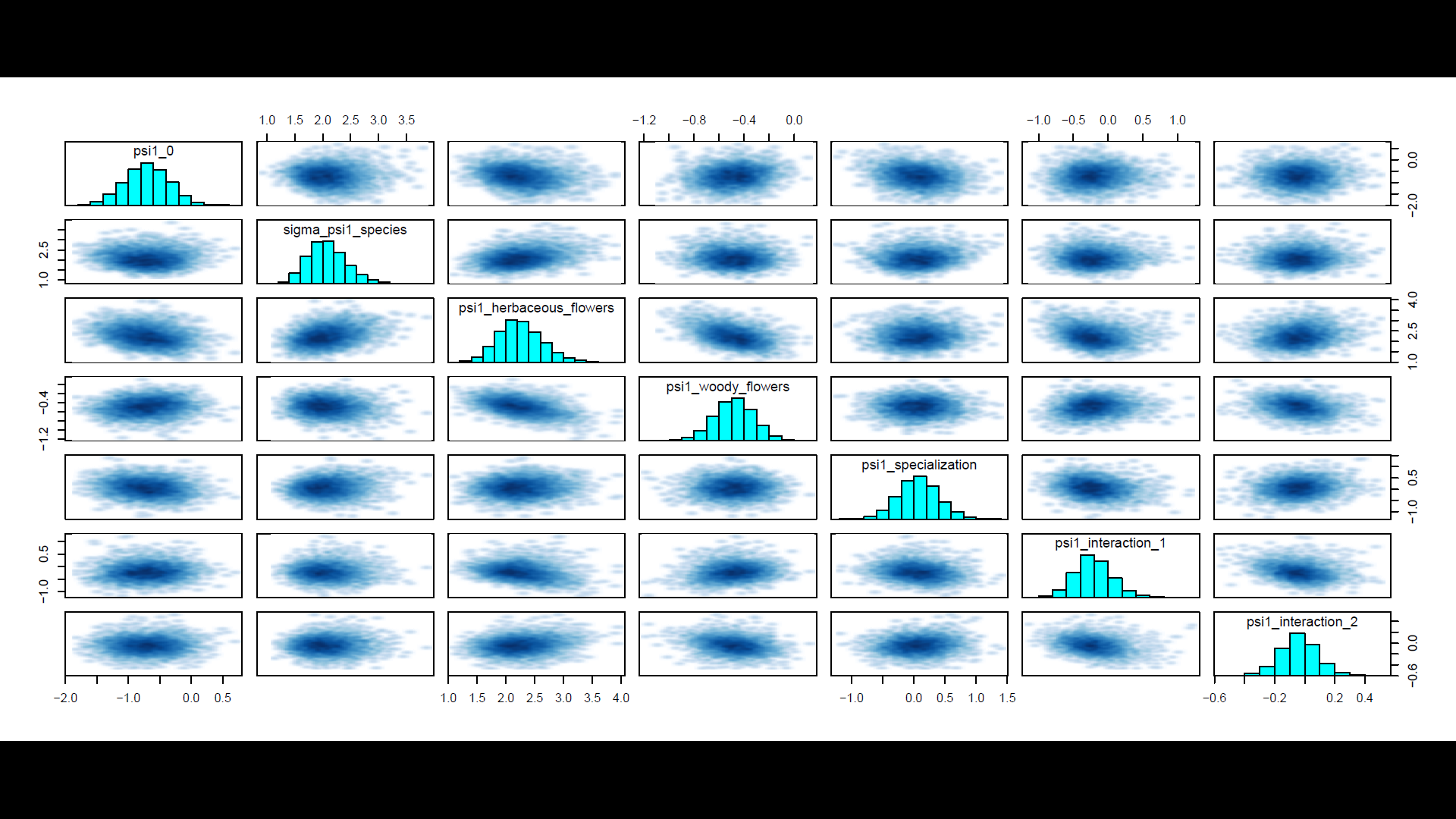
**Figure S6:** Pairs plot for occupancy model. Pairs plot for a sample subset of parameters (psi1). Lack of divergent transitions and no visual barriers in the parameter space search indicate full exploration of the posterior distribution.

**a)
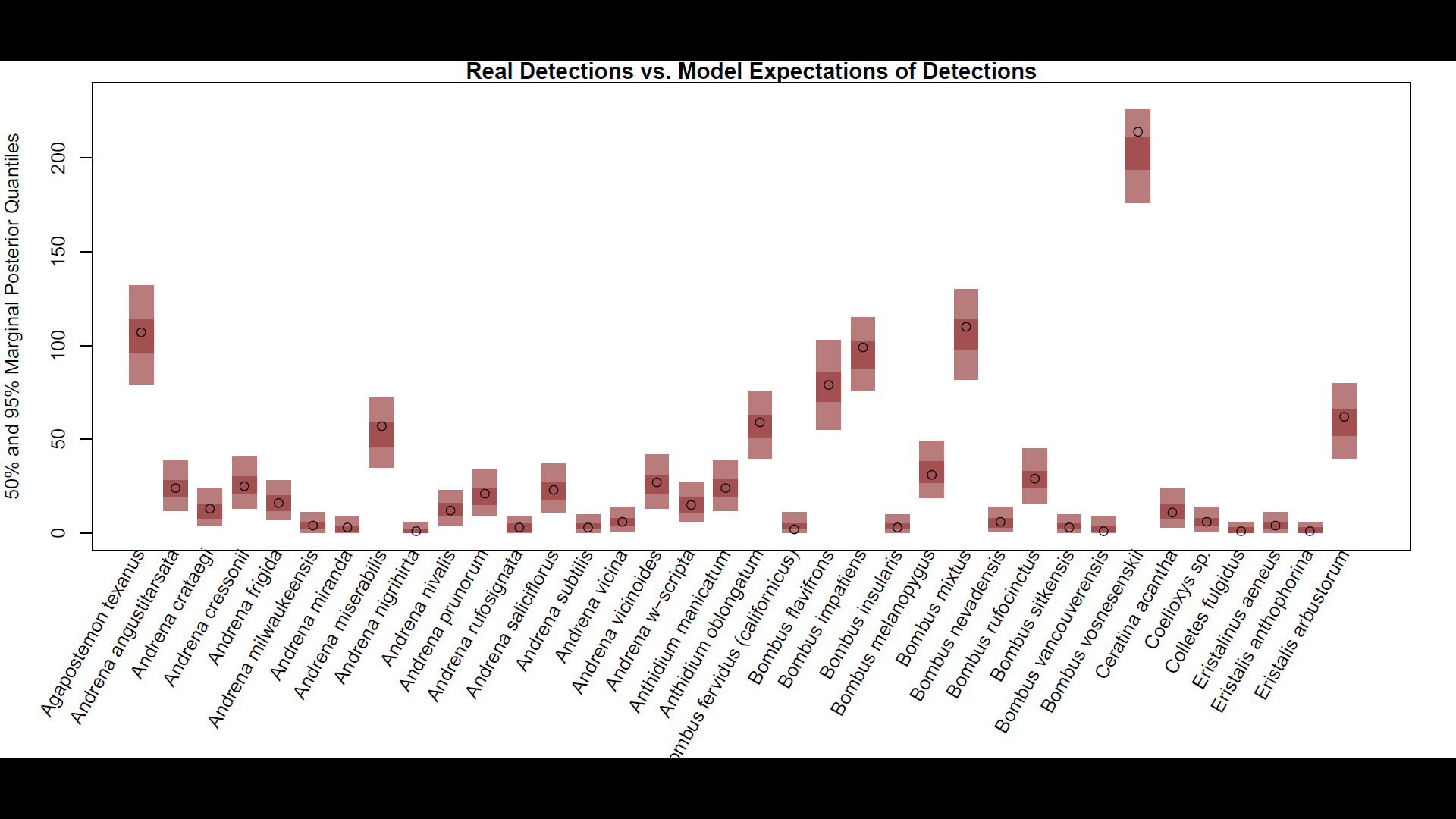
**

**b)
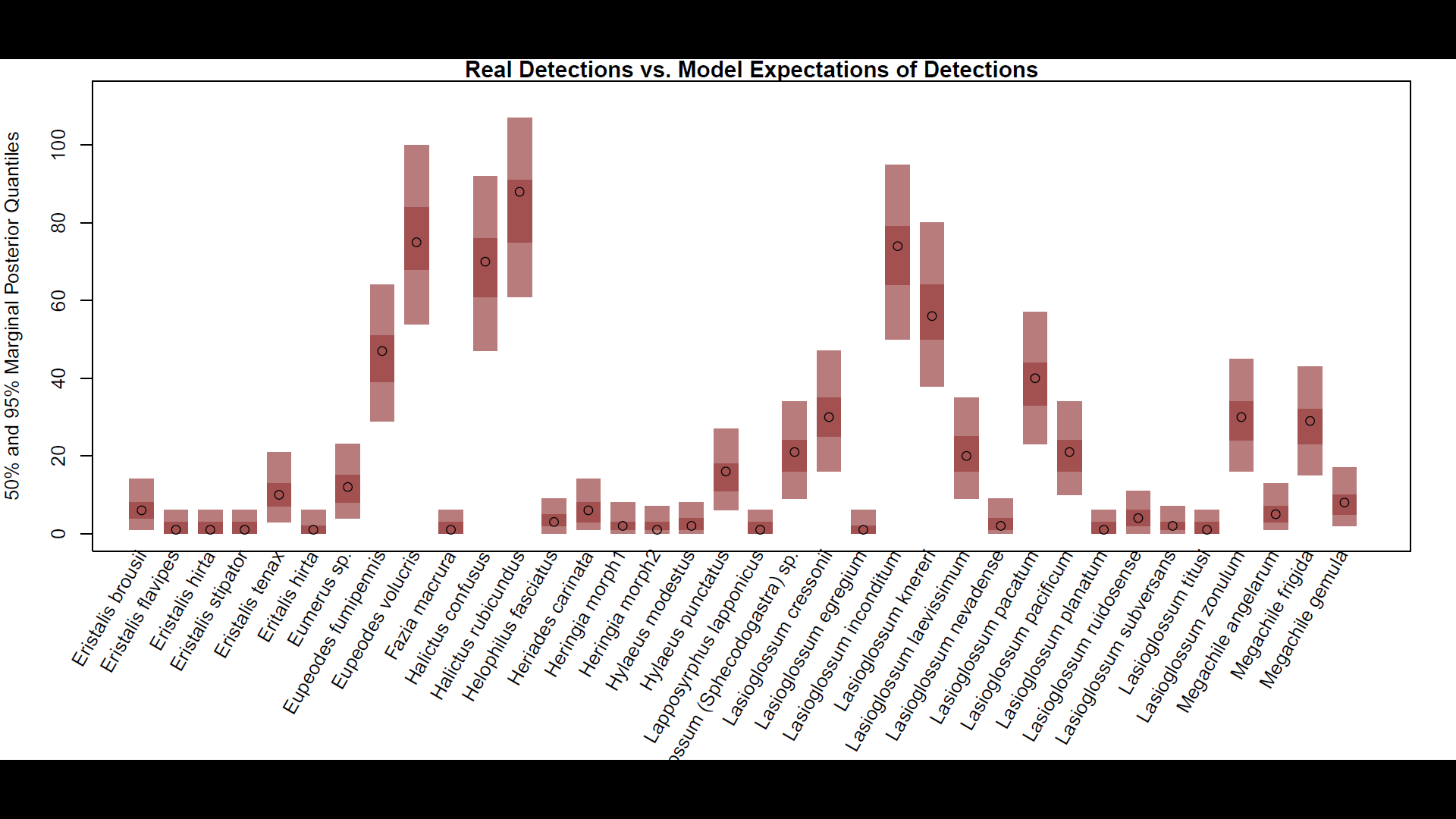
**

**c)**
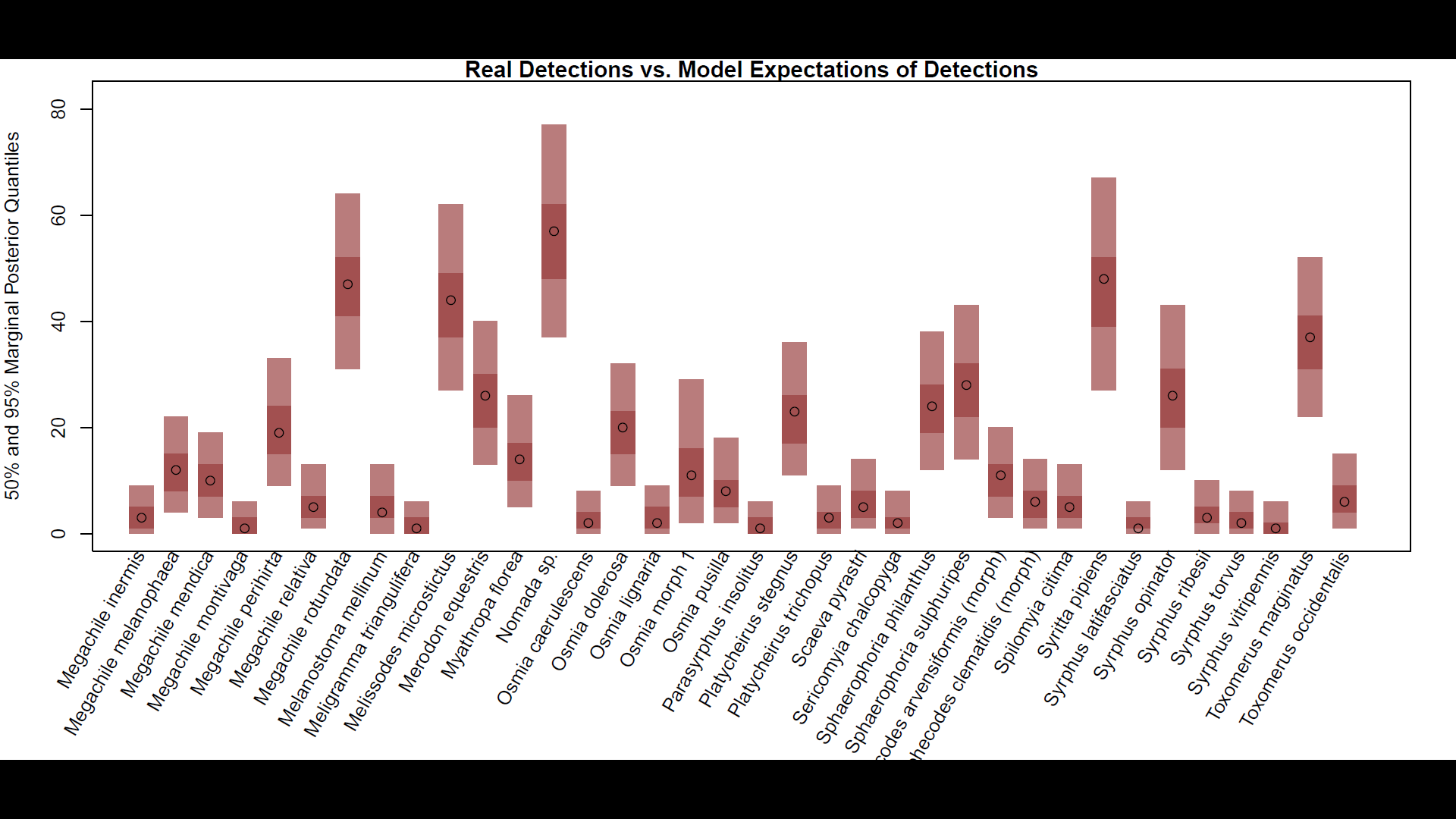


**Figure S7:** Posterior predictive check for occupancy model. At each step in the HMC run, we simulated the total number of detections across all visits for each species given the parameter estimates proposed by our model. After fitting the model, we visually compared the number of species-specific detections in our dataset to the posterior distribution of species-specific detections expected by the model. Correspondence between the observed number of detections (black circles) and the range of the number of detections predicted by the model (50% and 95% BCI’s in dark and light red) indicates a reasonable goodness-of-fit, i.e., the parameter values proposed by the model generate a realistic set of observations including accurately tracking the differences between species. Posterior predictive checks for the 108 species are broken into three panels (a – c), with species listed in alphabetical order.


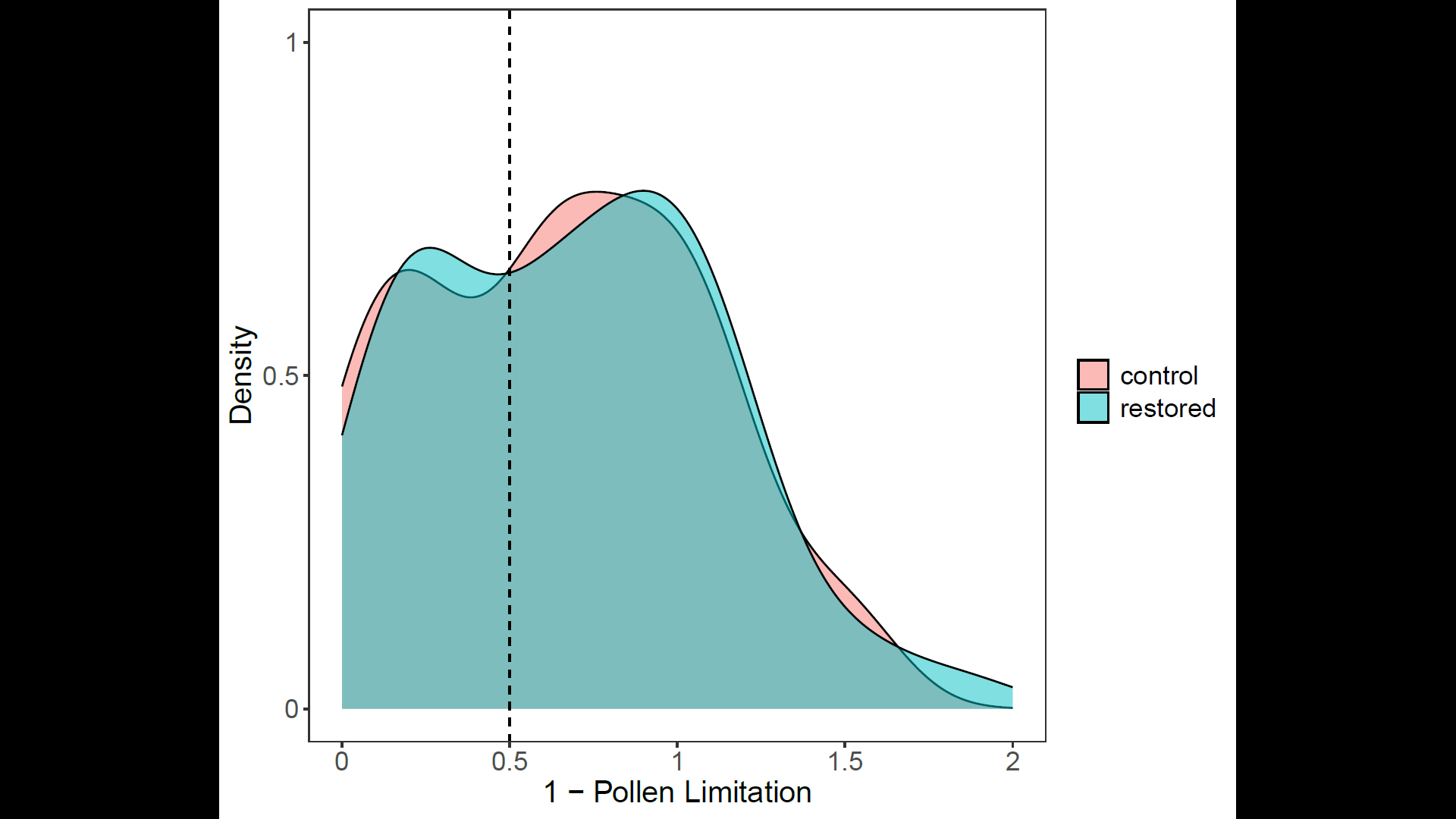


**Figure S8:** Raw pollen limitation rates. The complement of pollen limitation (1 - pollen limitation) was calculated by dividing seed set of a naturally pollinated flower by the seed set of a supplemented flower paired from the same plant and then subtracting the resulting value from 1. With this measure, a value of 1 indicates that a flower made a similar amount of seeds as a supplemented flower from the same plant; a value of 0.5 indicates that a flower made approximately half as many seeds as a supplemented flower from the same plant. The distribution of the compliment of pollen limitation is plotted for both control sites (red) and restored sites with herbaceous enhancement (blue). Because the distribution was bimodal and a continuous regression model resulted in poor model fit, we converted the response to a binary outcome, plants below the dashed line (0.5) were scored as pollen-limited versus plants above the dashed line scored as not pollen-limited. We also filtered out any plants for which naturally pollinated flowers made more than twice as many seeds as a supplemented flower as potential experimental errors.


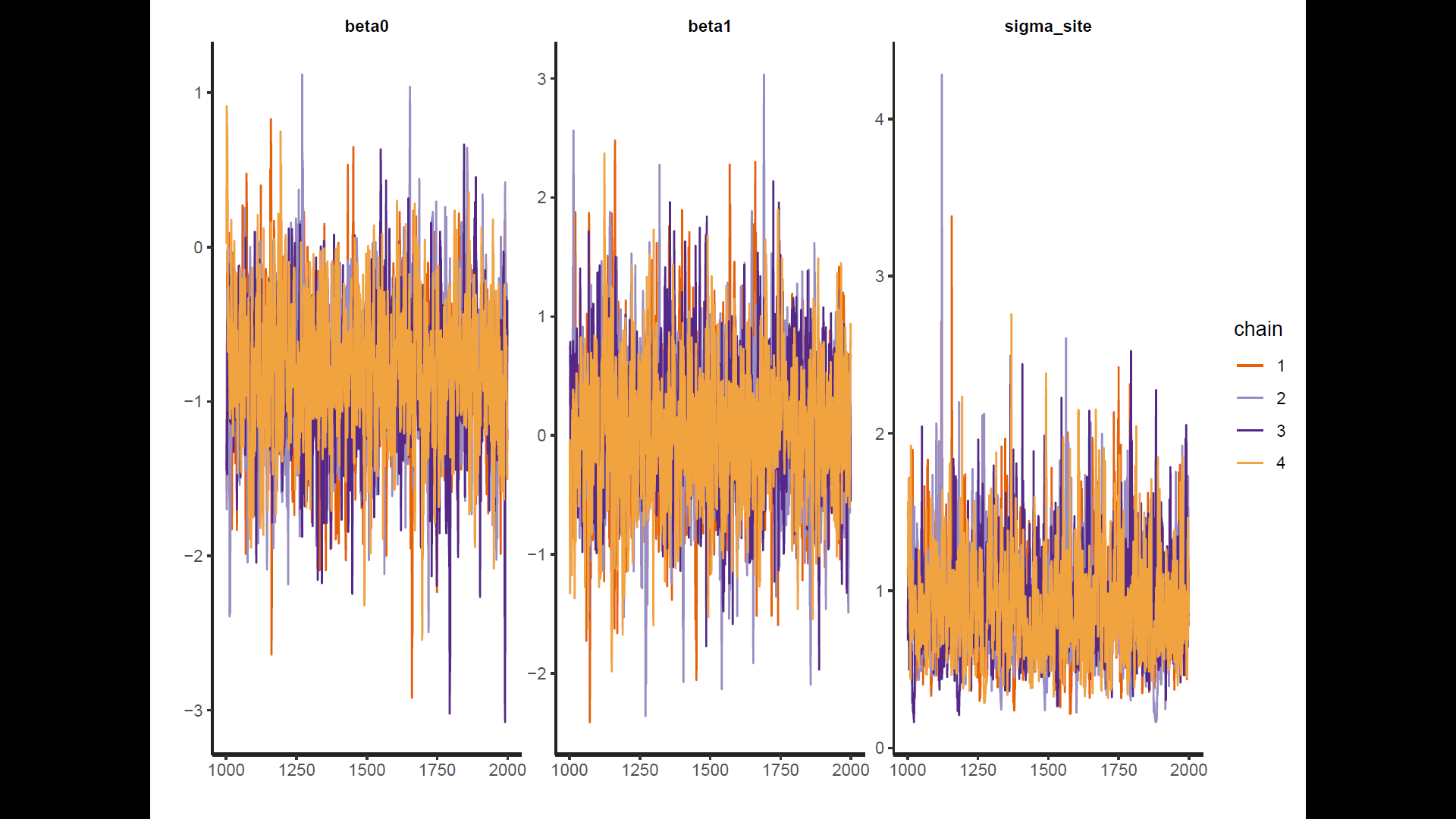


**Figure S9:** Traceplots and pairs plots for pollen limitation model. Overlapping HMC traces for all parameters indicate that all 4 chains have converged on a similar parameter space.


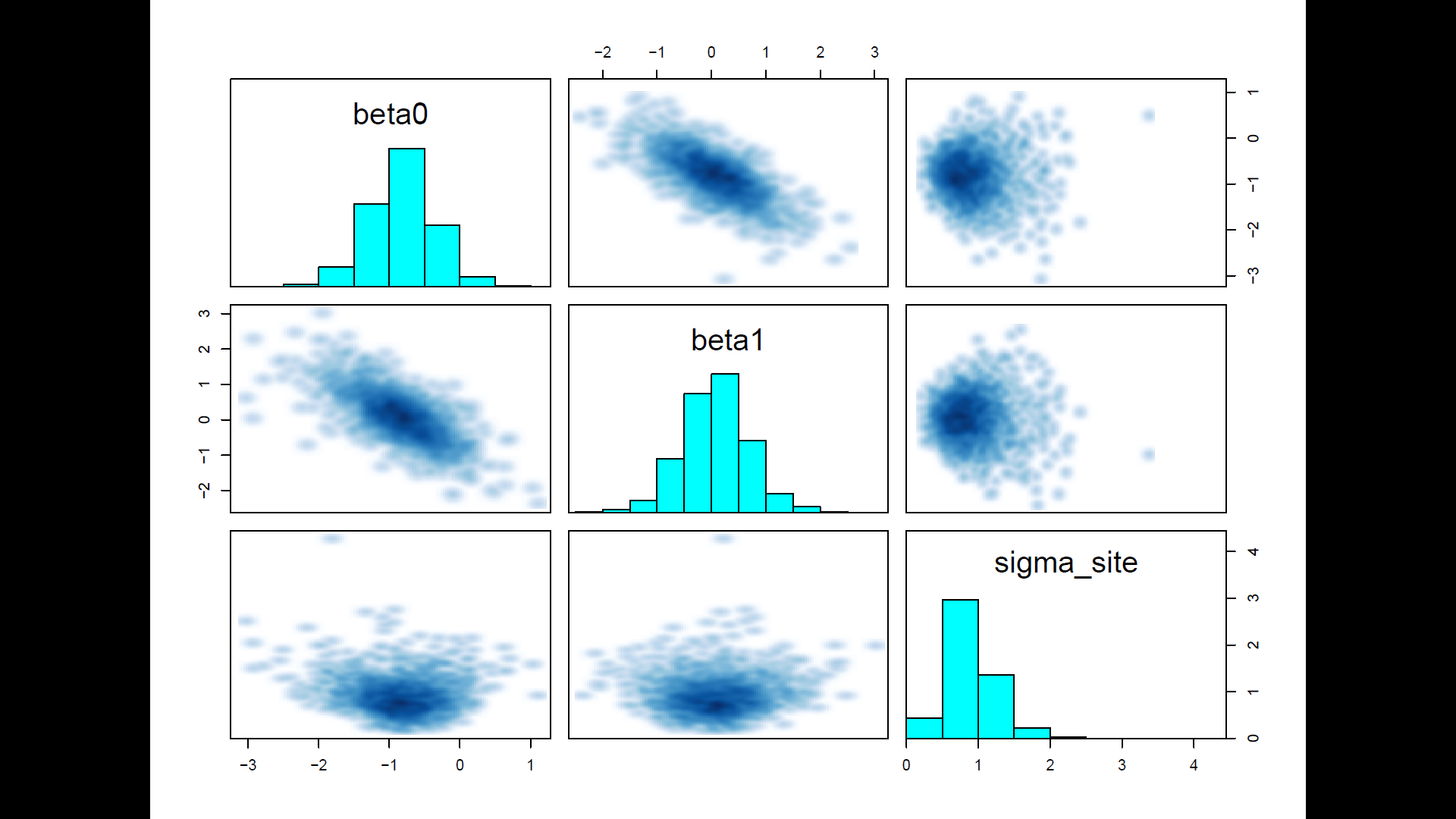


**Figure S10:** Pairs plot for pollen limitation model. Lack of divergent transitions and no visual barriers in the parameter space search indicate full exploration of the posterior distribution.


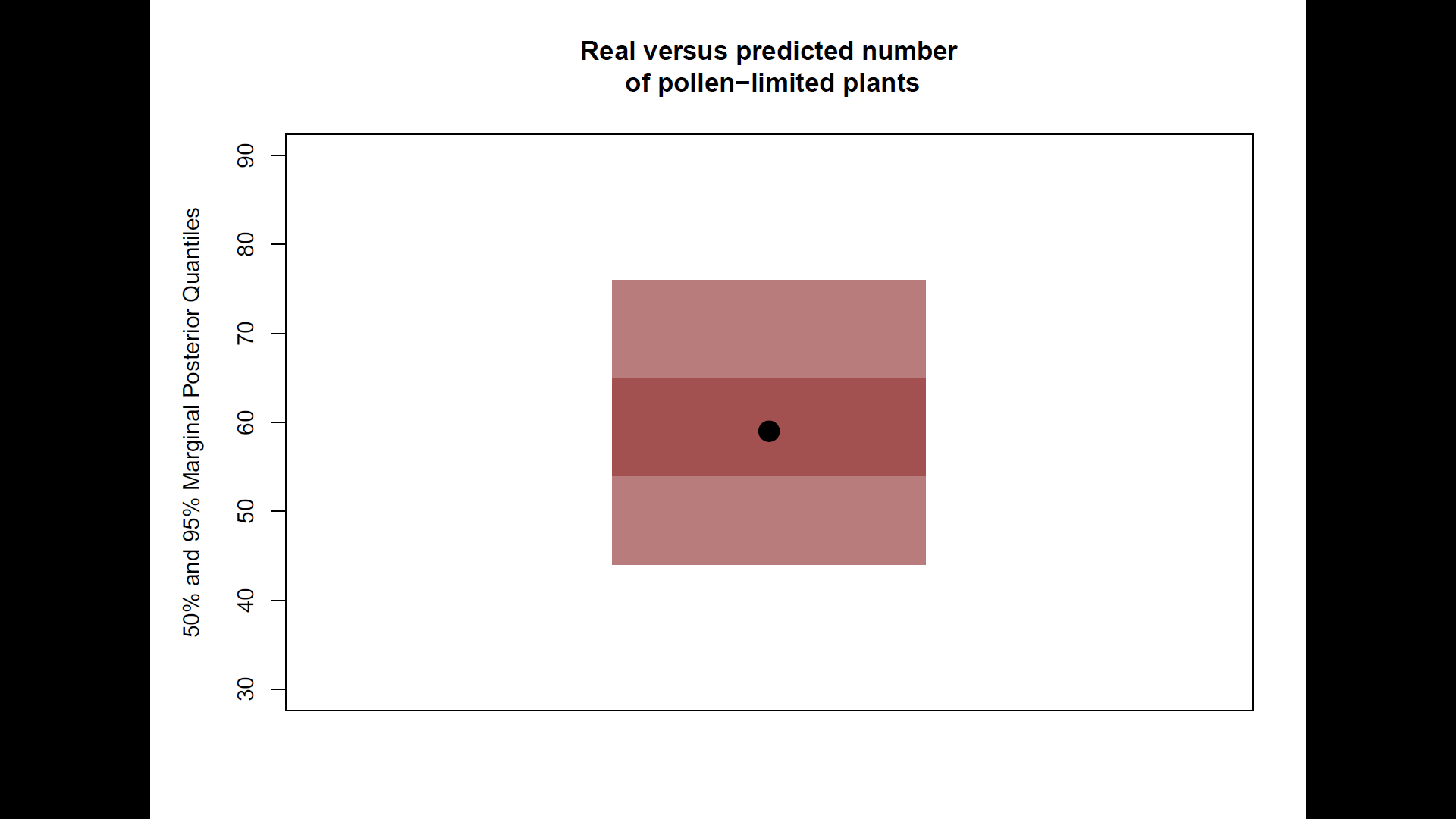


**Figure S11:** Posterior predictive check for pollen limitation model. Number of pollen limited plants (out of 175) falling within the range of the number of pollen limited plants (out of 175) predicted by the model indicate a reasonable goodness-of-fit, i.e., the model parameter values proposed by the model can generate a realistic set of observations.


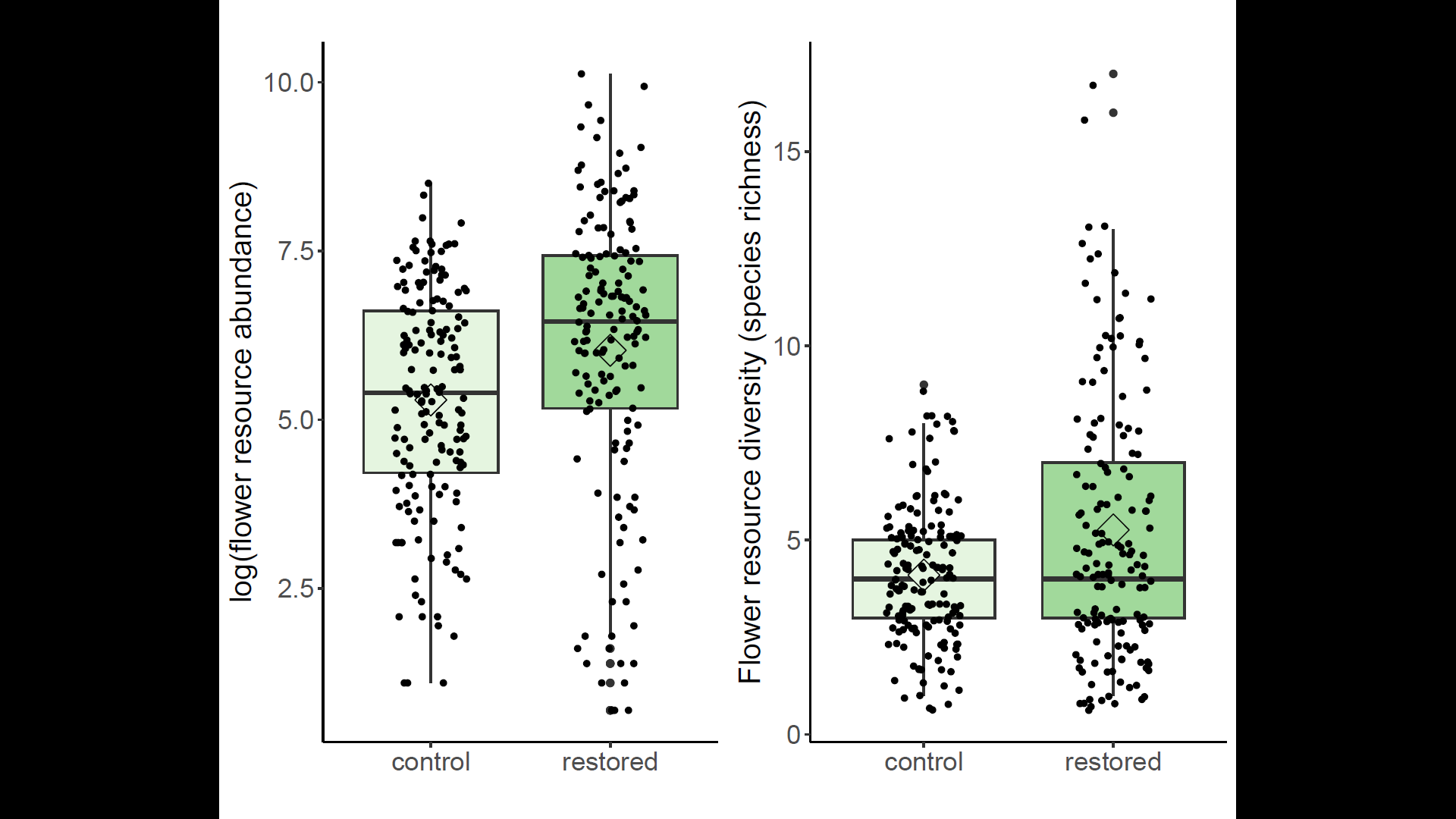


**Figure S12:** Herbaceous plant floral abundance and diversity (excluding plant species visited by pollinators on <5 occasions) in control versus restored sites. Jittered points represent survey specific values of abundance and diversity, boxplot midlines indicate group medians while diamonds indicate group means. We compared floral abundance and diversity using prepackaged Bayesian GLM’s implemented through rstanarm (Figure S13). Light green indicates control sites and dark green indicates restored sites.

1.
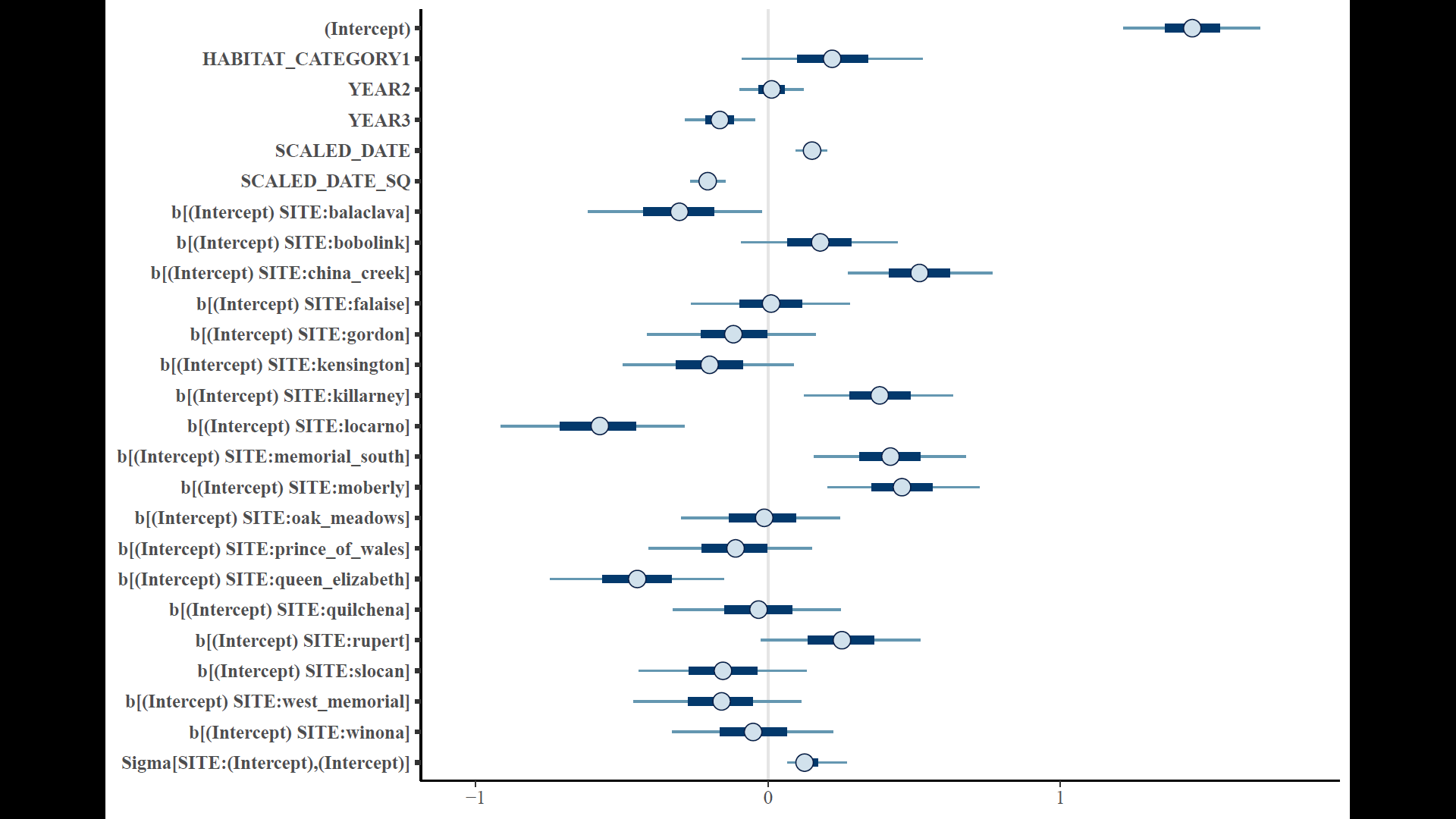

2.
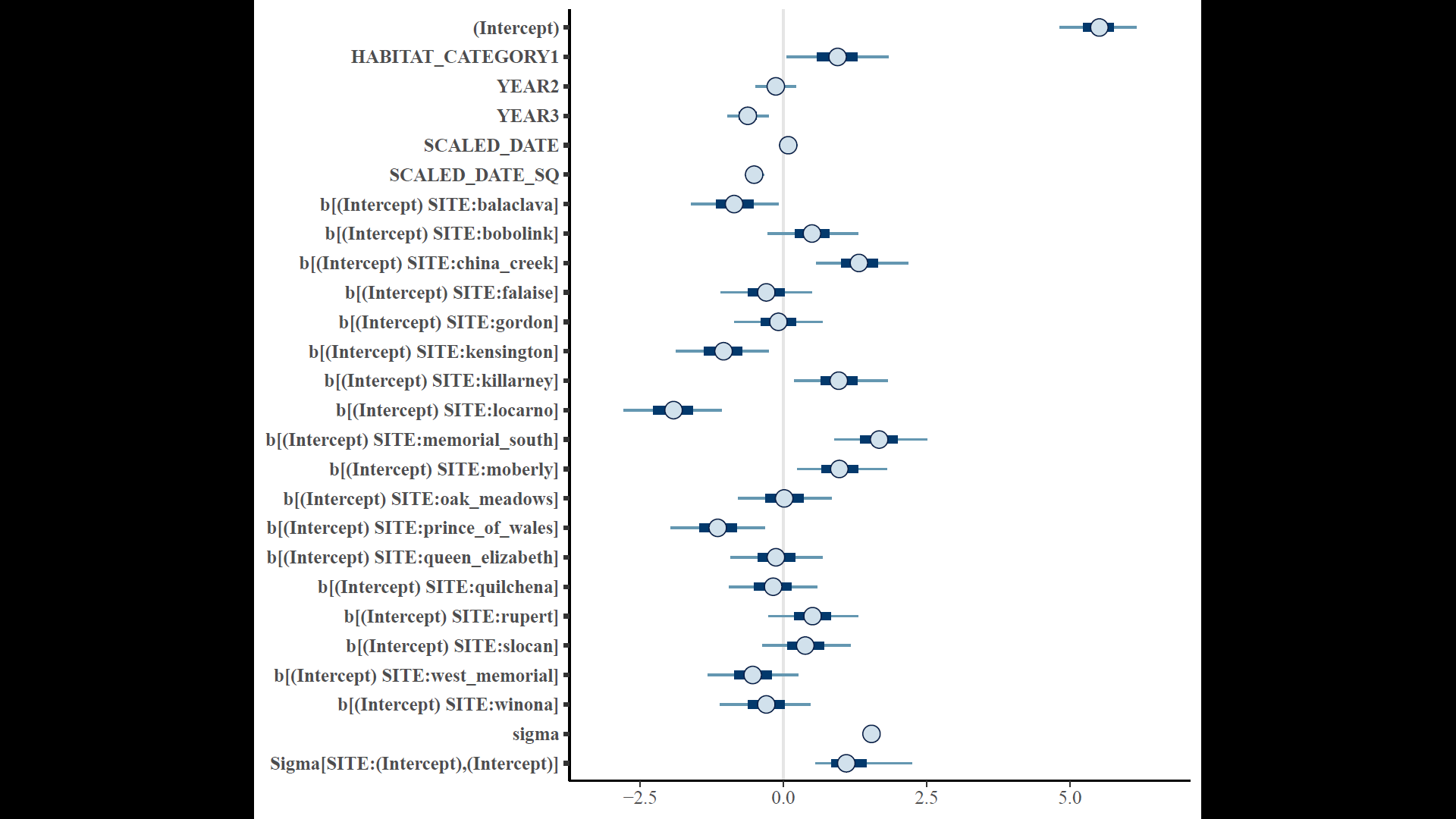


**Figure S13:** Model estimates for the effect of herbaceous enhancement on survey-specific flower abundance and richness (excluding plant species visited by pollinators on <5 occasions). We compared flower abundance using a poisson model and found a marginal positive effect of restoration (50% BCI for “habitat category” > 0) (a). We compared flowering plant species richness using a poisson model and found a strong positive effect of restoration (95% BCI for “habitat category” > 0) (b). For both models we included potential effects of year, day of year, day of year squared and also a random effect for site to account for repeated measures.


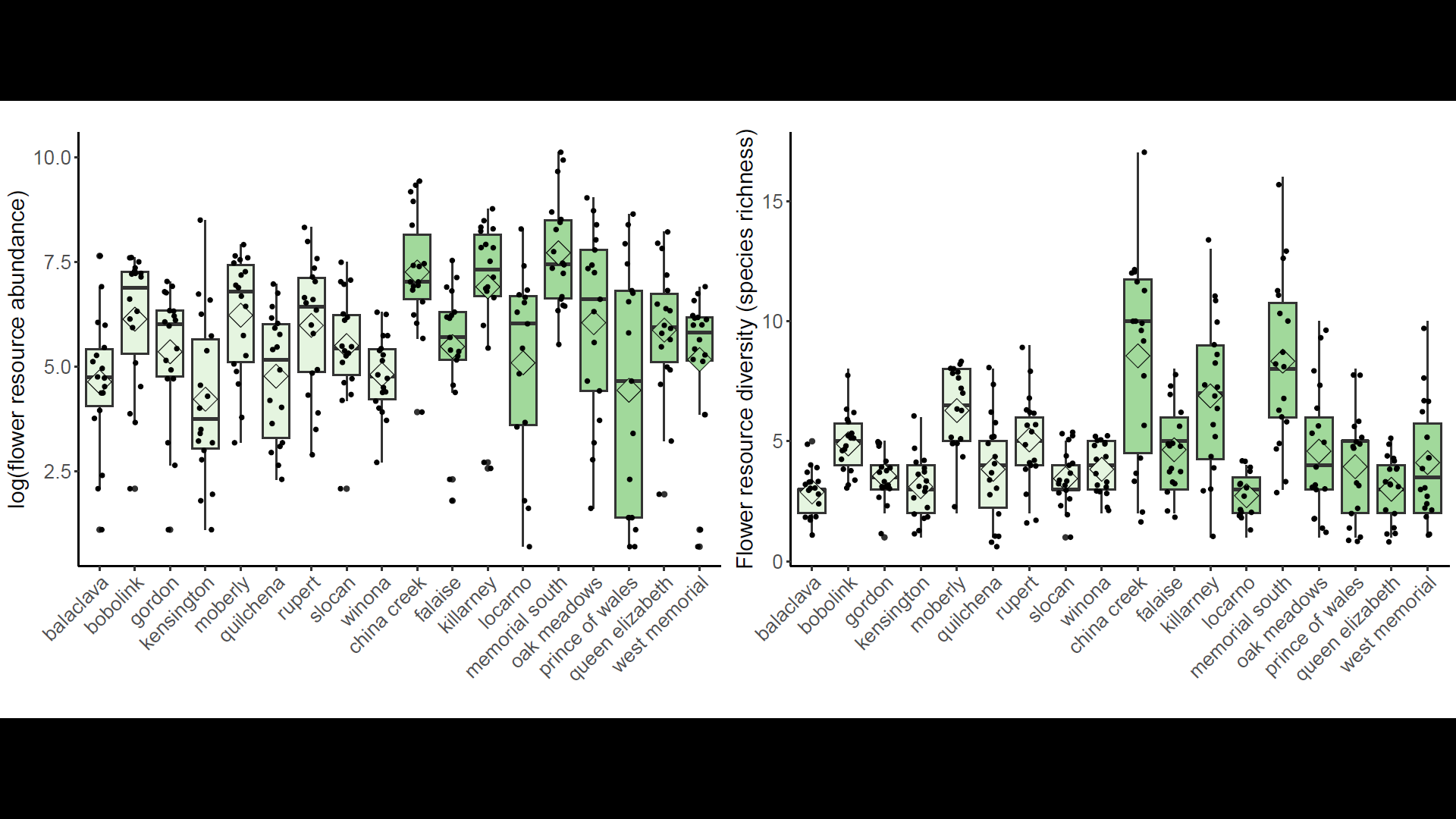
**Figure S14:** Among-site variation in herbaceous floral resources (excluding plant species visited by pollinators on <5 occasions). Jittered points represent survey specific values of abundance and diversity, boxplot midlines indicate group medians while diamonds indicate group means. Light green indicates control sites and dark green indicates restored sites.


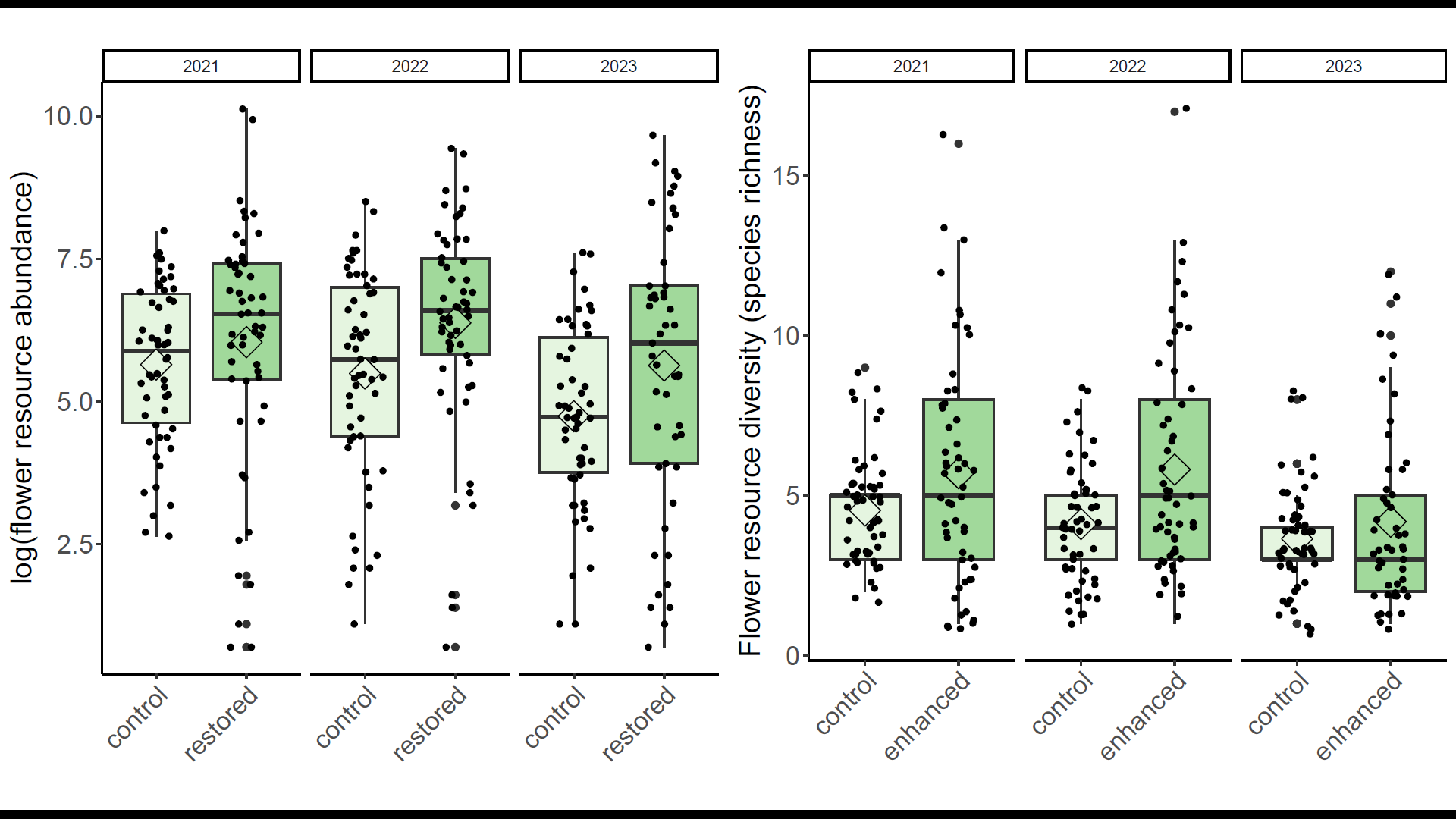


**Figure S15:** Annual variation in herbaceous floral resources (excluding plant species visited by pollinators on <5 occasions). Jittered points represent survey specific values of abundance and diversity, boxplot midlines indicate group medians while diamonds indicate group means. Light green indicates control sites and dark green indicates restored sites.

1.
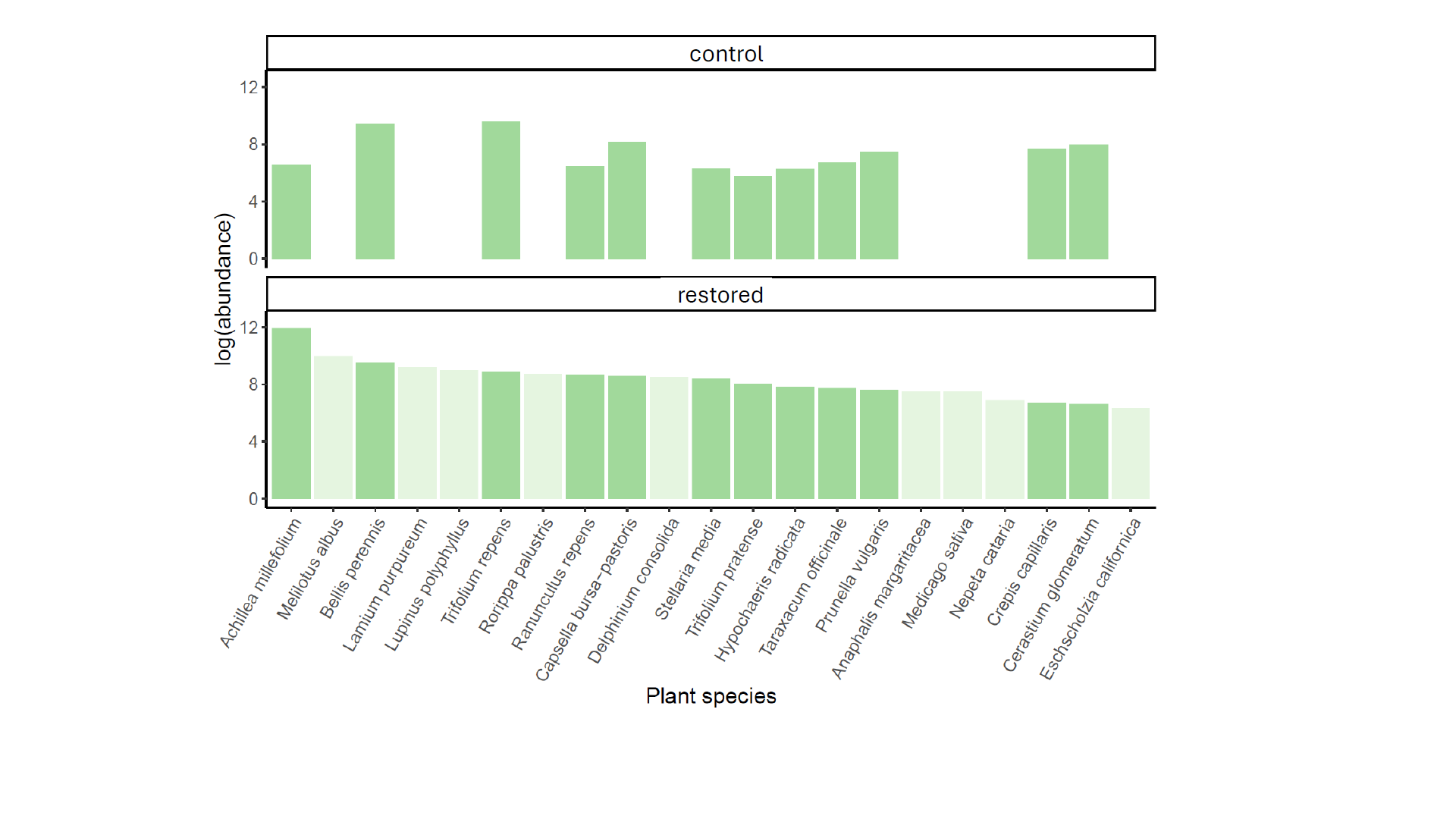

2.
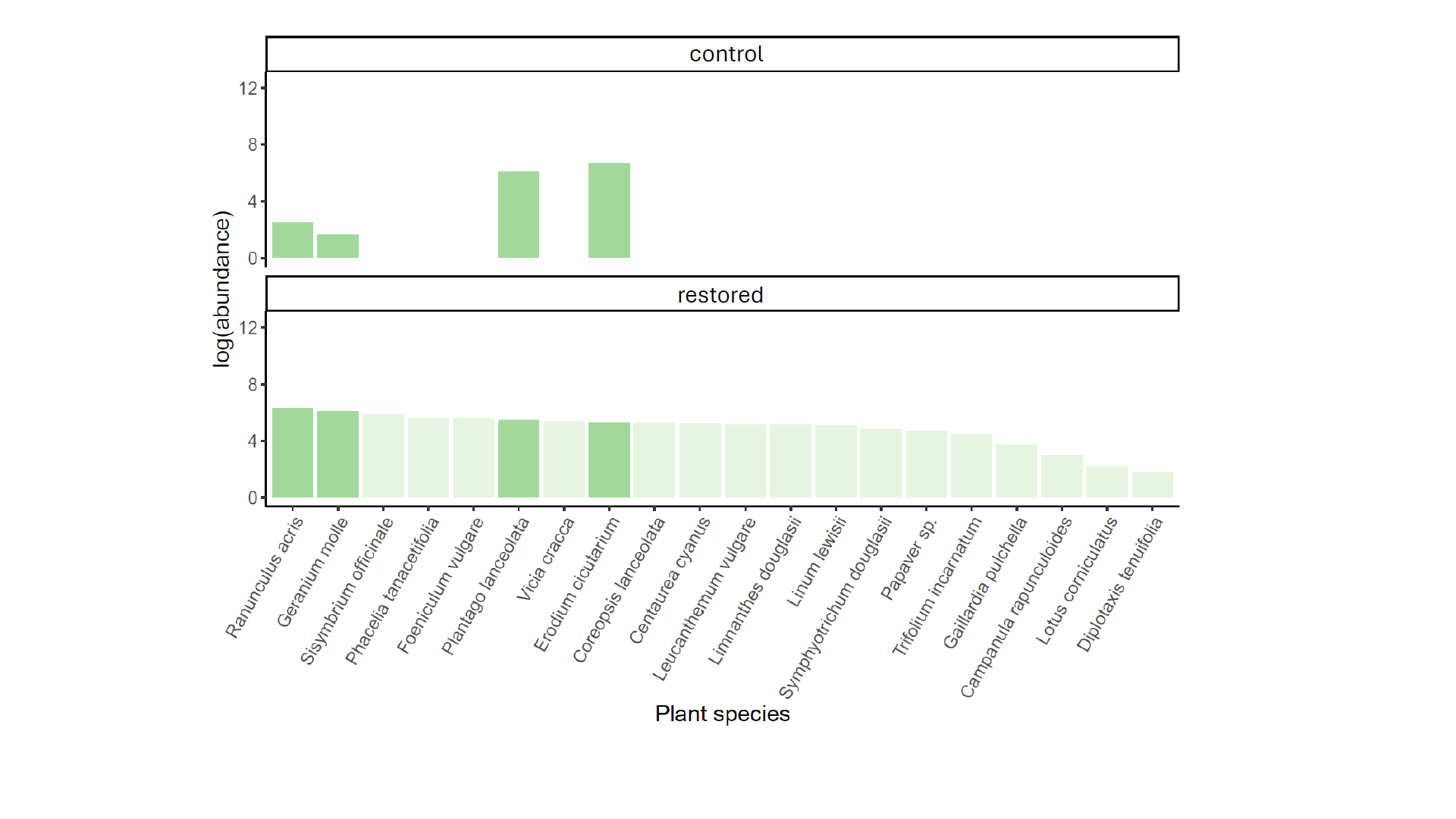


**Figure S16:** Herbaceous flower composition in restored sites enhancements (below) versus control (above) (excluding plant species visited by pollinators on <5 occasions). Y-axis indicates the log(total abundance) observed across all quadrat surveys across all parks within each category. Plants are listed in order of total abundance observed across all sites, separated into panels (a and b) for visualization. Dark green bars indicate species that occurred at both control sites and restored sites. Light green bars indicate species that occurred only at restored sites. No species unique to control parks were recorded, meaning that the control site diversity was a nested subset of the restored site diversity.


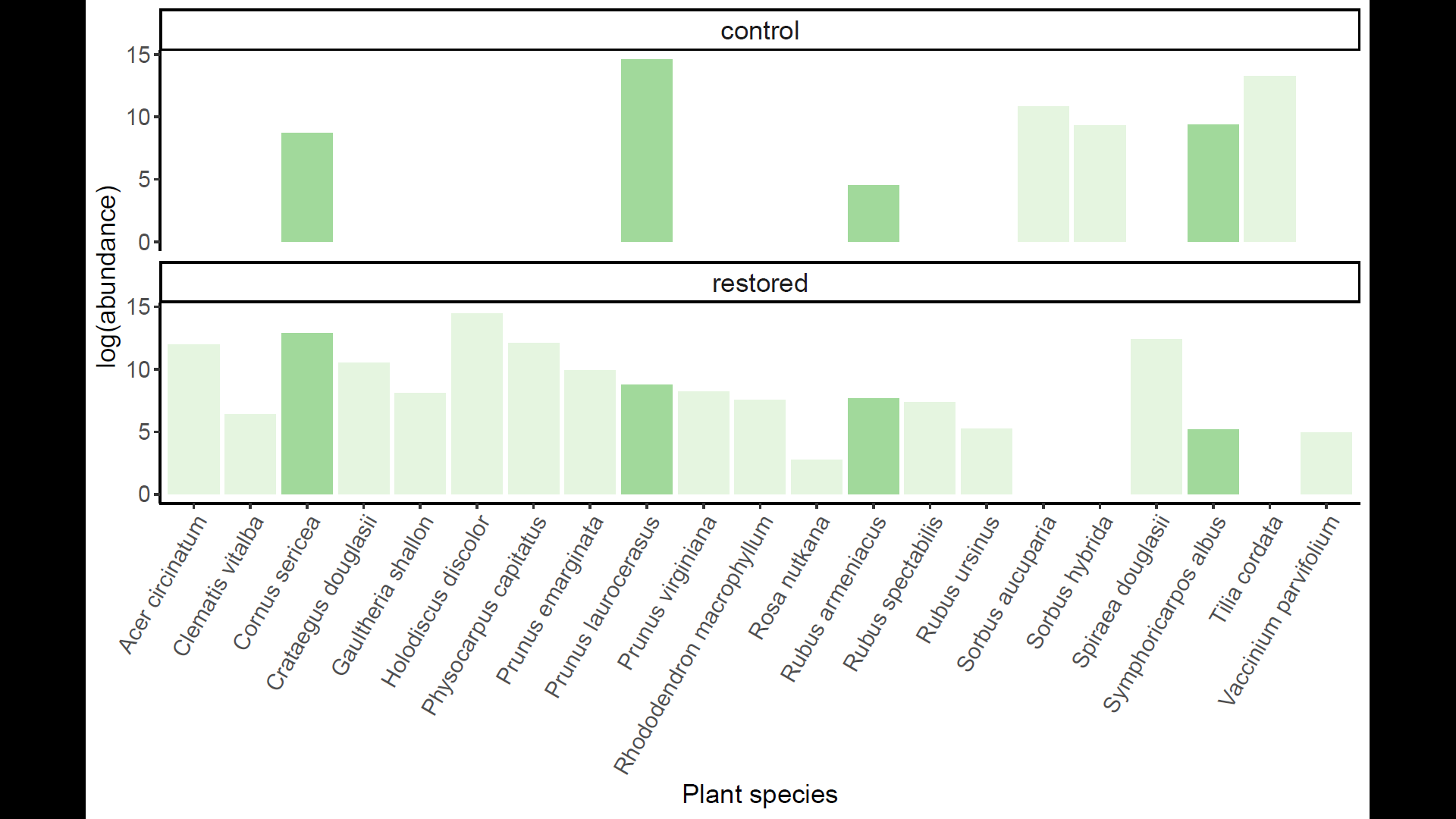


**Figure S17:** Woody flower composition in sites restored with herbaceous enhancements (below) versus control (above) (excluding plant species visited by pollinators on <1 occasions). Y-axis indicates the log(total abundance) observed across all woody plant surveys across all parks within each category. Dark green bars indicate species that occurred at both control sites and restored sites. Light green bars indicate species that occurred only at one site type.

1.
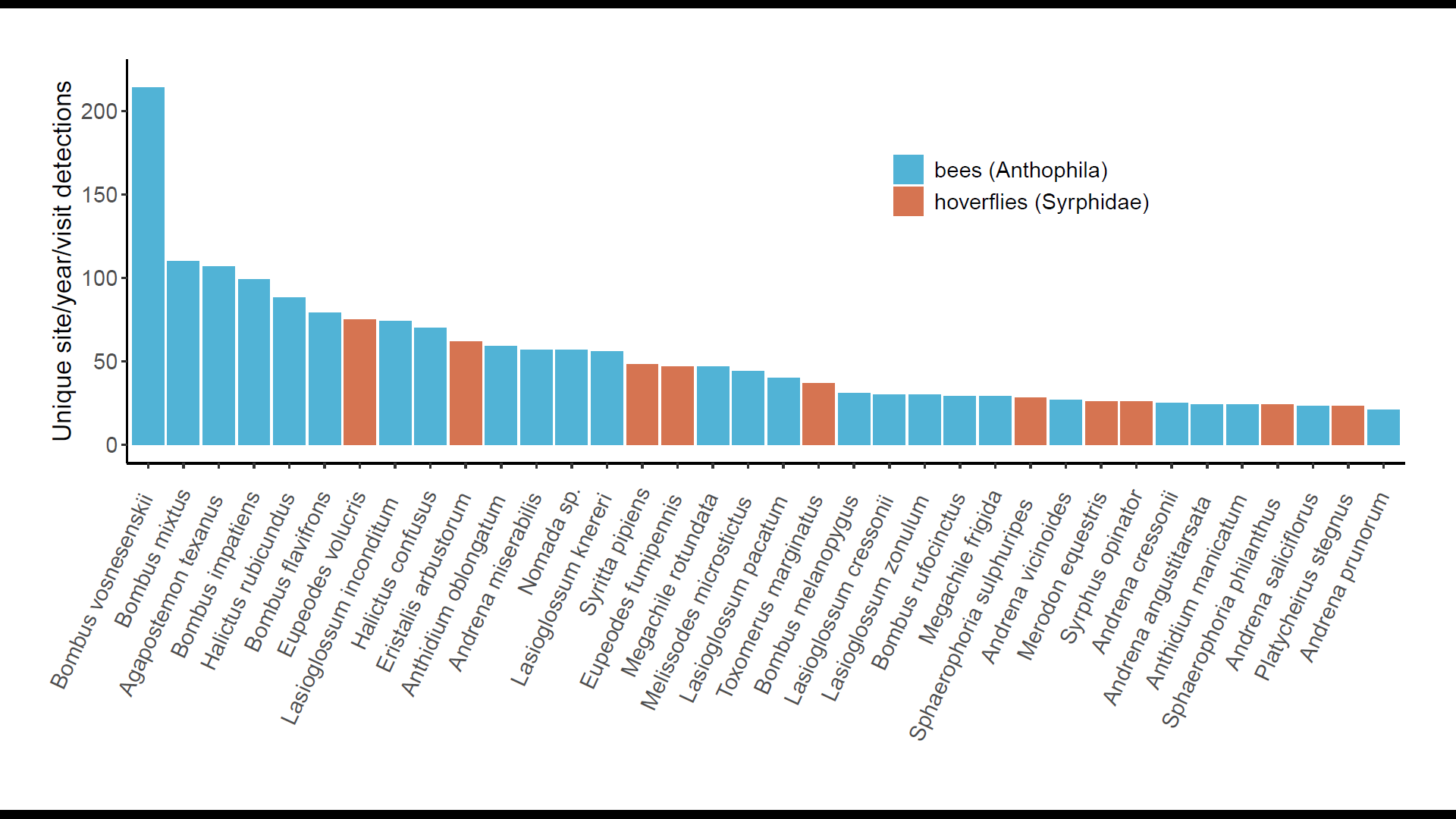

2.
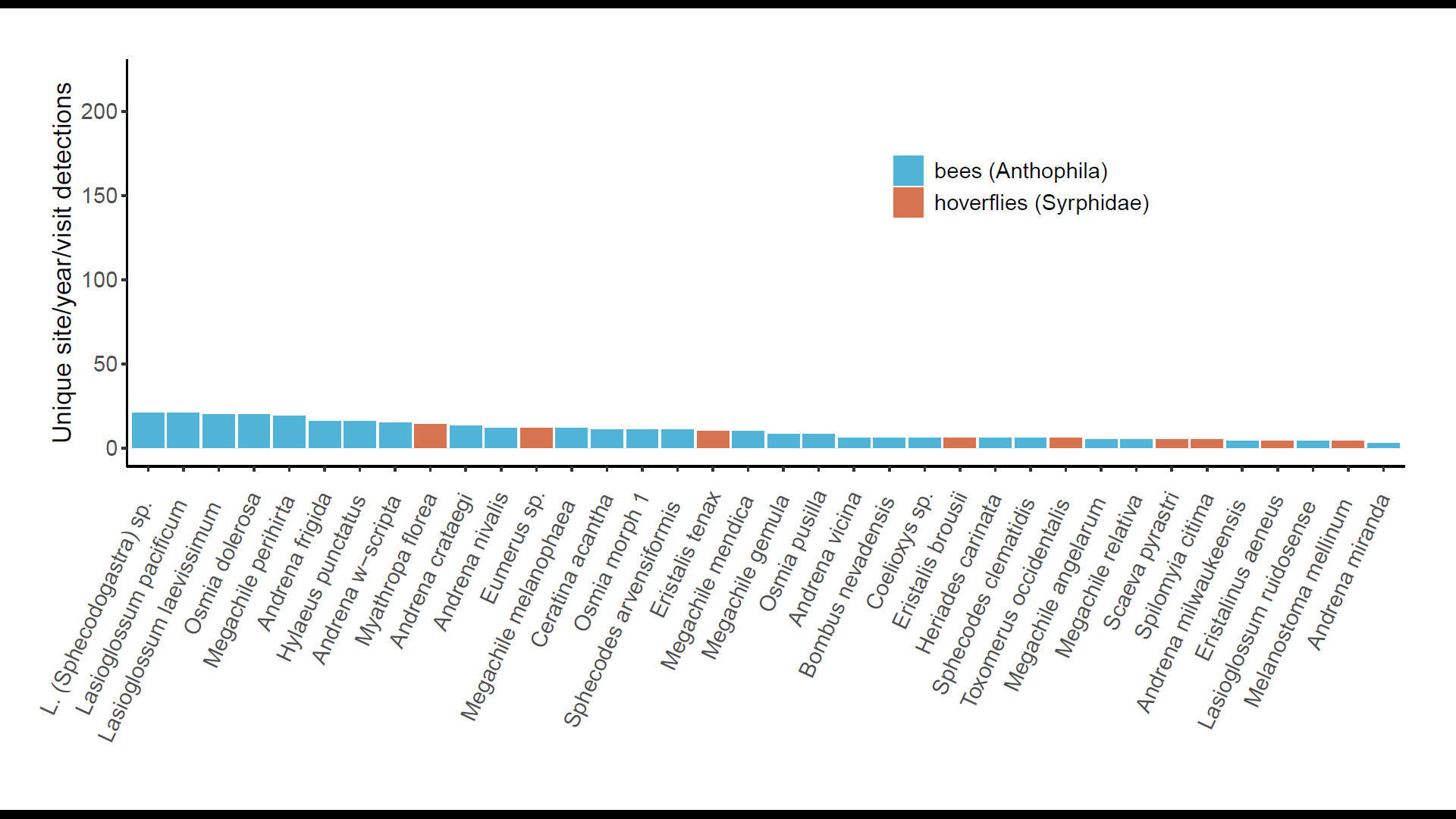

3.
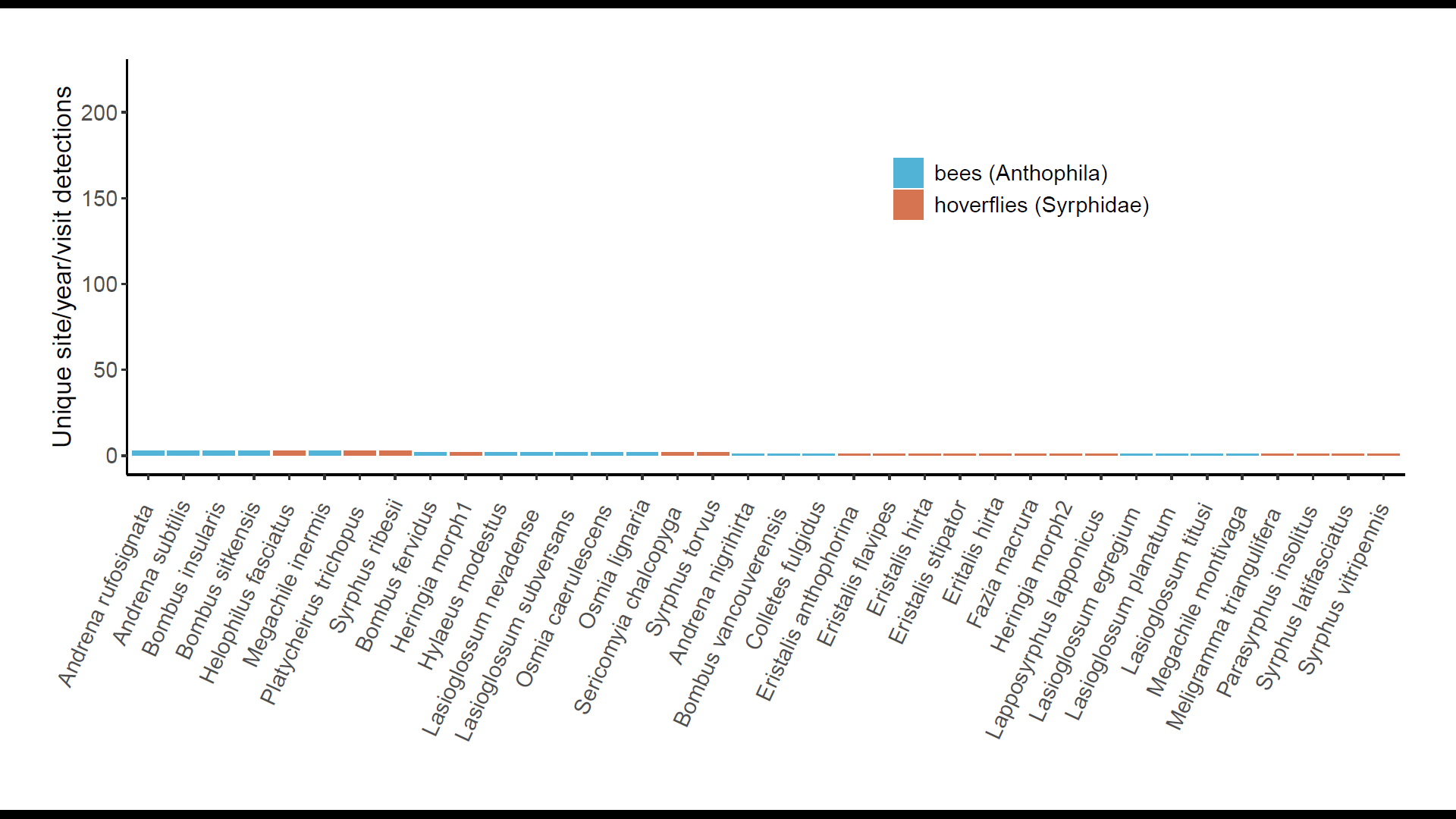


**Figure S18:** Total unique pollinator detections by species. Detection counts are displayed for the most common species in our study (species detected 5 or more times). Additional species were detected on fewer occasions and still included in our models. Honey bee (*Apis mellifera*) detections are reported here, however, we did not include honey bees in our analyses.

1.
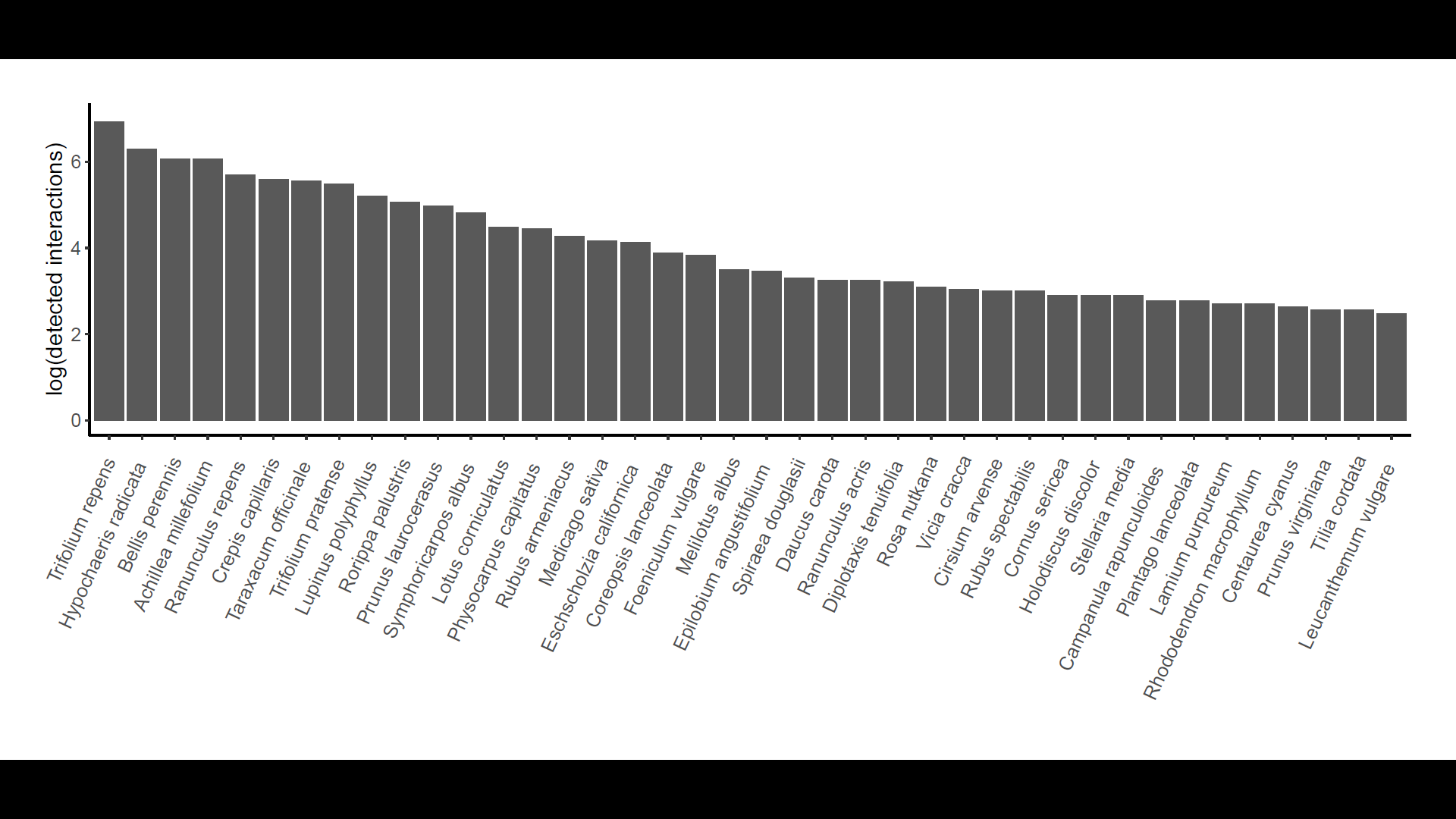

2.
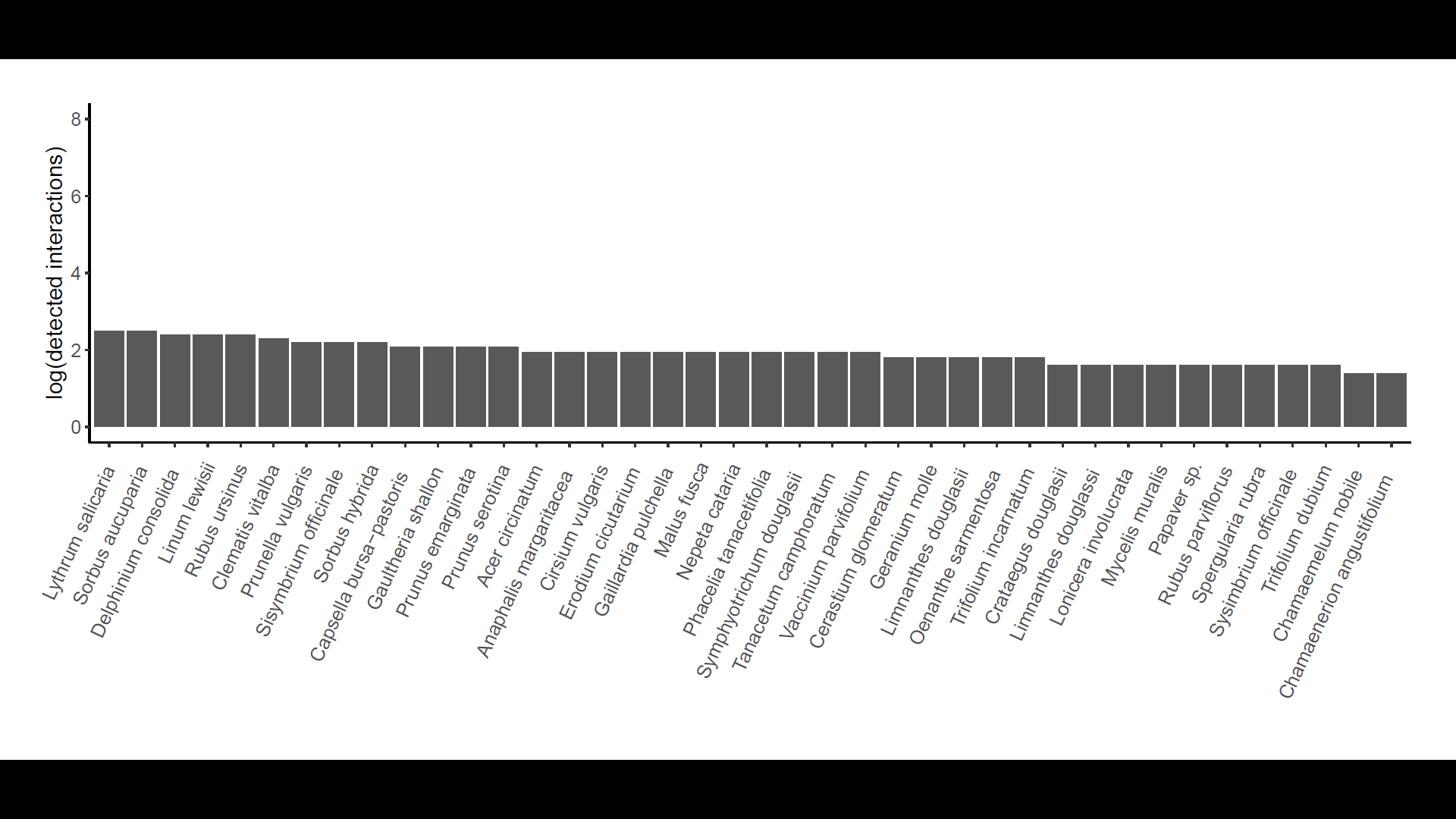

3.
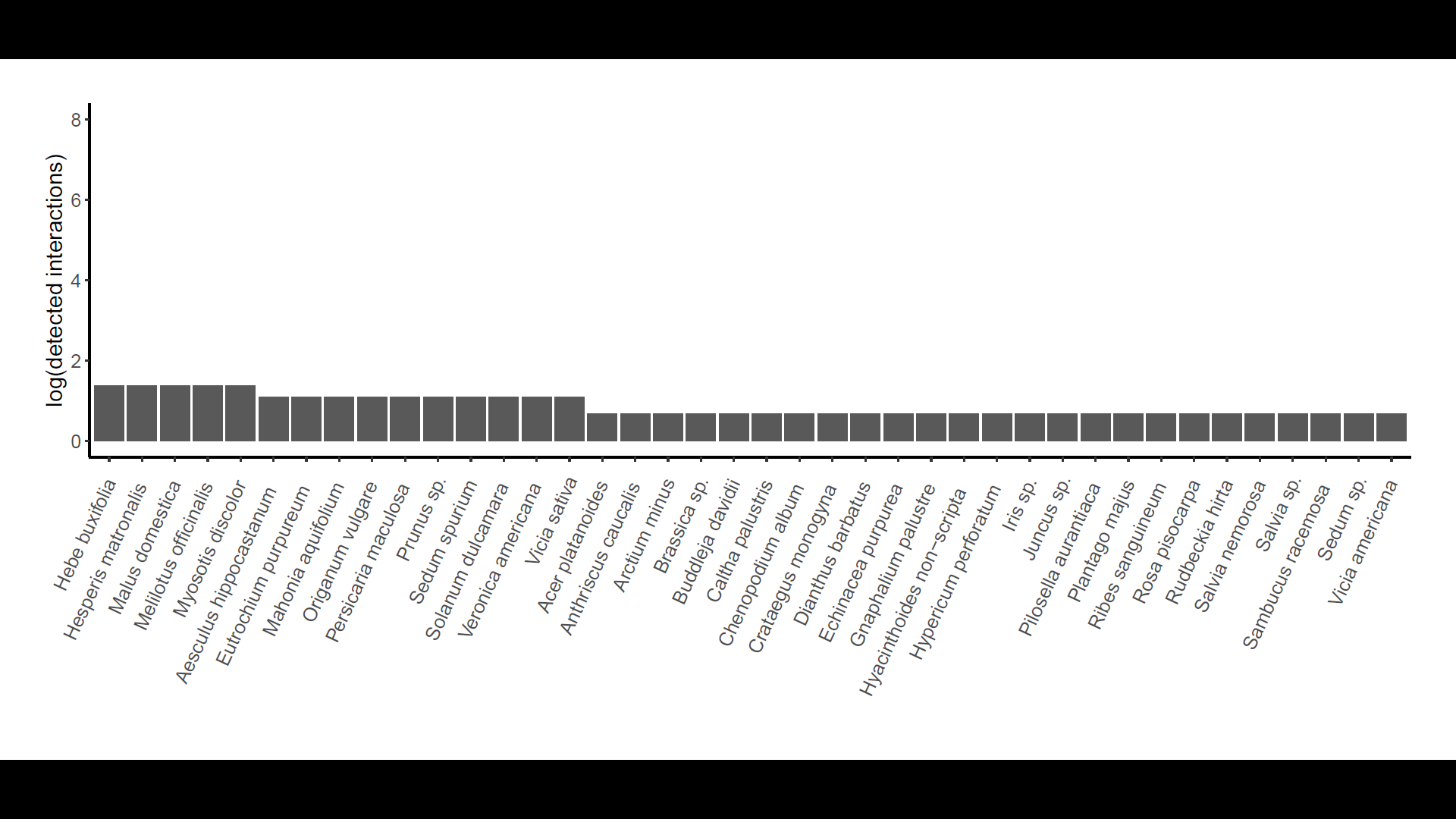


**Figure S19:** (Log-scaled) frequency of interactions observed during our field surveys. Species are listed in order of total number of interactions, split across panels (a – c). Supplemented by interactions from external studies, we used these interactions to estimate network-based species-specific pollinator specialization metrics.


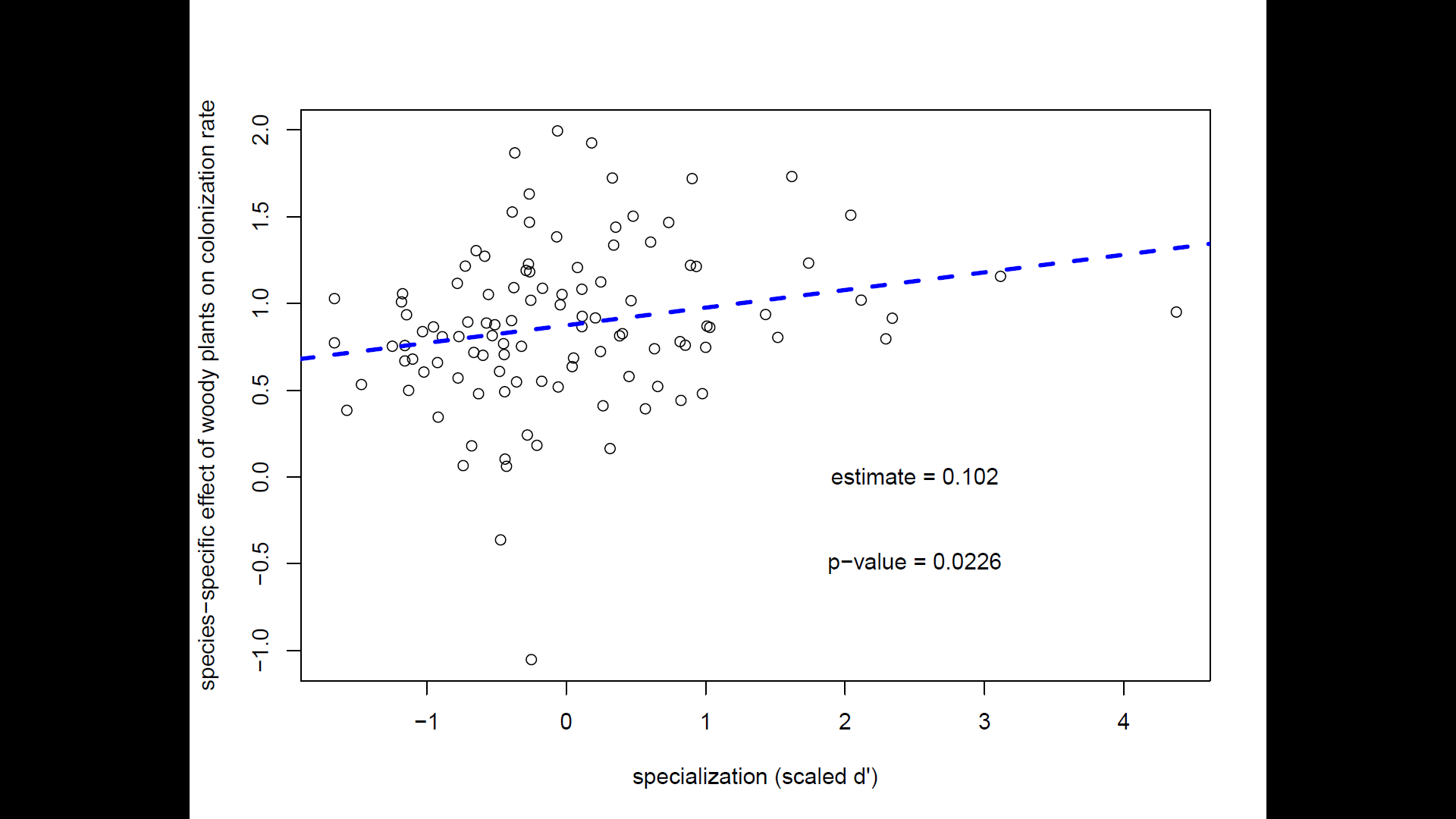


**Figure S20:** *A posteriori* assessment of specialization. We re-fit our model using species-specific random effects rather than effects of specialization and interactions between specialization and restoration or woody plants. We then tested whether specialization was a significant predictor of any of the six random-effects of interest, species-specific effects of: restoration on initial occurrence, restoration on colonization, restoration on persistence, woody plants on initial occurrence, woody plants on colonization, and woody plants on persistence. Specialization was only a significant predictor (p-value < 0.05) of species-specific effects of woody plants on colonization (data and association shown in Figure S20). This is consistent with our original *a priori* findings that specialization only strongly interacts with the effects of woody plants on colonization rate. Notably, this *a posteriori* assessment suggests that colonization rates were only negatively effects by woody plants for two species (points falling below zero on the y-axis): *Eupeodes volucris* and *Lasioglossum knereri*.


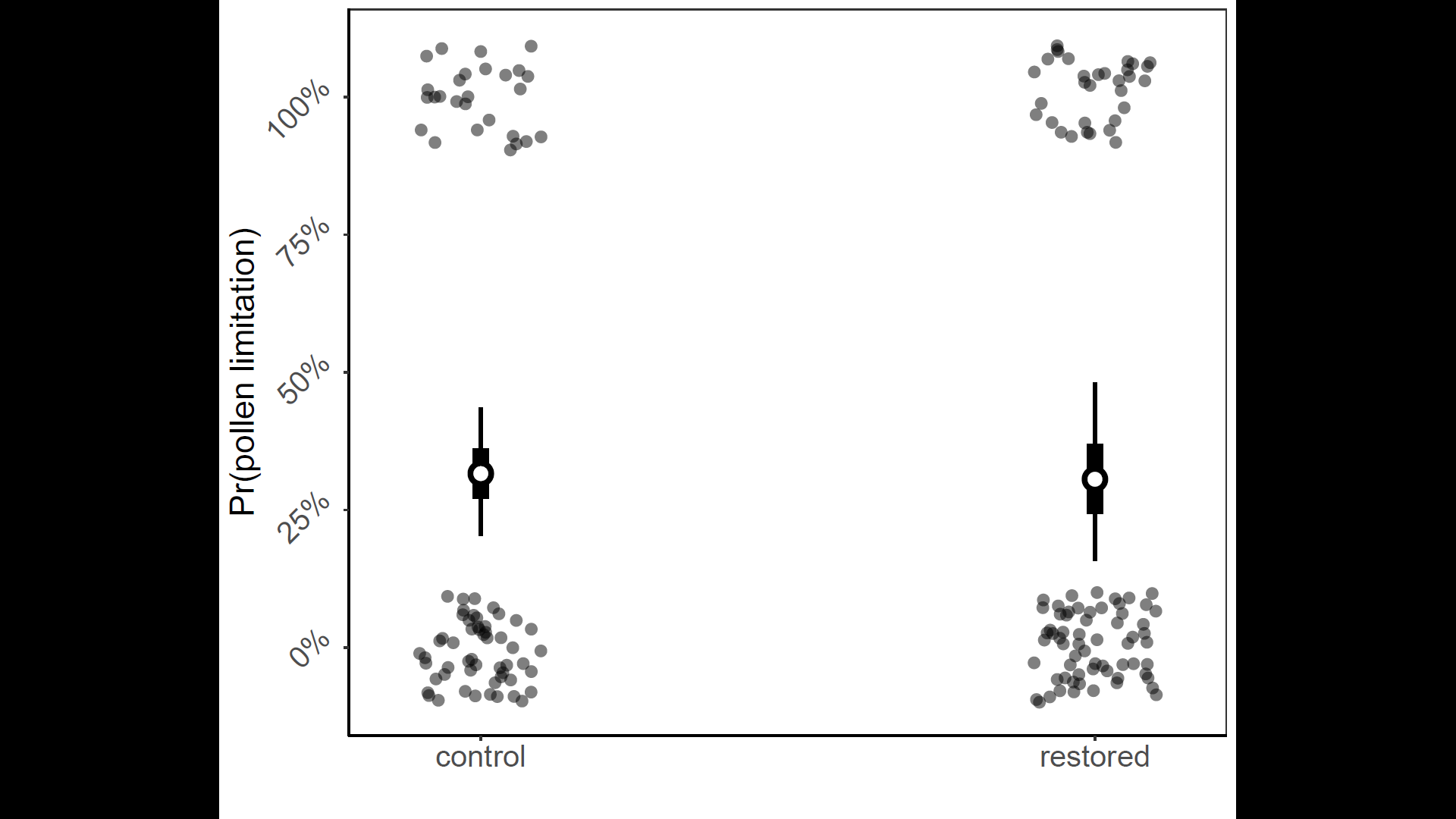


**Figure S21:** We compared the seed set of the fruit capsule from the pollen supplemented flower against the seed set for a fruit capsule developing from a paired, neighboring flower from the same inflorescence that developed under ambient pollination conditions. Comparison flowers that produced fewer than 50% of seeds relative to the pollen supplemented flower on the same plant were scored as pollen limited. We then tested the association between whether a site was restored with an herbaceous enhancement and the probability of pollen limitation (c). Dark and light grey uncertainty bands indicate 50% BCI and 95% BCI’s. Jittered points show the observed pollen limitation outcomes for the 175 plants from 11 sites included in the experiment, where 0 indicates no pollen limitation and 1 indicates strong pollen limitation.
